# Lack of genomic evidence of AI-2 receptors suggests a non-quorum sensing role for *luxS *in most bacteria

**DOI:** 10.1186/1471-2180-8-154

**Published:** 2008-09-20

**Authors:** Fabio Rezzonico, Brion Duffy

**Affiliations:** 1 Agroscope Changins-Wädenswil ACW, Division of Plant Protection, CH-8820 Wädenswil, Switzerland

## Abstract

**Background:**

Great excitement accompanied discoveries over the last decade in several Gram-negative and Gram-positive bacteria of the LuxS protein, which catalyzes production of the AI-2 autoinducer molecule for a second quorum sensing system (QS-2). Since the *luxS *gene was found to be widespread among the most diverse bacterial taxa, it was hypothesized that AI-2 may constitute the basis of a universal microbial language,  a kind of bacterial Esperanto. Many of the studies published in this field have drawn a direct correlation between the occurrence of the *luxS *gene in a given organism and the presence and functionality of a QS-2 therein. However,  rarely hathe existence of potential AI-2 receptors been examined. This is important,  since it is now well recognized that LuxS also holds a central role as a metabolic enzyme in the activated methyl cycle which is responsible for the generation of S-adenosyl-L-methionine,  the major methyl donor in the cell.

**Results:**

In order to assess whether the role of LuxS in these bacteria is indeed related to AI-2 mediated quorum sensing we analyzed genomic databases searching for established AI-2 receptors (i.e.,  LuxPQ-receptor of *Vibrio harveyi *and Lsr ABC-transporter of *Salmonella typhimurium*) and other presumed QS-related proteins and compared the outcome with published results about the role of QS-2 in these organisms. An unequivocal AI-2 related behavior was restricted primarily to organisms bearing known AI-2 receptor genes,  while phenotypes of *luxS *mutant bacteria lacking these genes could often be explained simply by assuming deficiencies in sulfur metabolism.

**Conclusion:**

Genomic analysis shows that while LuxPQ is restricted to Vibrionales,  the Lsr-receptor complex is mainly present in pathogenic bacteria associated with endotherms. This suggests that QS-2 may play an important role in interactions with animal hosts. In most other species,  however,  the role of LuxS appears to be limited to metabolism,  although in a few cases the presence of yet unknown receptors or the adaptation of pre-existent effectors to QS-2 must be postulated.

## Background

Population density- and growth phase-dependent bacterial cell-to-cell communication involving the production and detection of extracellular signaling molecules (autoinducers) is referred to as quorum sensing (QS). The detection of a stimulatory concentration of an autoinducer leads to an alteration in gene expression,  enabling bacteria to coordinate their behavior in response to environmental challenges. A large number of cellular functions are known to be regulated via the QS mechanism in a variety of bacterial species such as biofilm formation,  swarming behavior or the production of antibiotics and virulence factors [1]. *N*-acyl-homoserine lactones (AHLs) are the most frequent signaling molecules found in Gram-negative bacteria. This first autoinduction system (QS-1) was identified in the luminescent marine symbiont *Vibrio fischeri *and is based on the pheromone *N*-3-oxohexanoyl-L-homoserine lactone [2,3]. Meanwhile several other type I autoinducers (AI-1),  whose molecules differ only in the AHL-acyl side chain moiety,  have been discovered in a number of Gram-negative bacteria [1]. Since every species employs specific receptors for its own AHL molecule,  not every bacterium can automatically cross-talk with all other bacteria,  so that every 'diAHLect' can essentially be understood only by those species sharing the same cognate receptor. However,  due to the fact that different bacteria may simultaneously use several signal molecules and receptors,  and given the chemical similarity of the different AHL molecules,  a limited interaction (e.g.,  attenuated response or interference) is possible even for bacteria carrying non-cognate receptors. Thus,  a limited (but not universal) communication between different bacterial species is possible through QS-1 [4-6].

In this respect great interest accompanied the discovery,  first in *Vibrio harveyi *[7] then in a variety of other bacteria [8],  of the LuxS protein that catalyzes the production of an autoinducer molecule (AI-2) for a second quorum sensing system (QS-2) [9]. Since the *luxS *gene was subsequently found to be widespread among both Gram-negative and Gram-positive bacteria,  it was tempting to presume that QS-2 may constitute the basis of an universal language,  a sort of bacterial Esperanto [10]. The presence of *luxS *has been reported in several subgroups of the bacterial kingdom,  being widely found in Bacteroidetes,  Actinobactetria,  and β- plus γ-Proteobacteria,  along with all Bacilli,  Deinococci and ε-Proteobacteria,  but not in Archaea or Eukarya [11]

In *Vibrio *species QS-2 is connected with QS-1 by partially sharing the same transduction pathway leading through the central signal relay protein LuxU to the terminal response regulator LuxO [12,13],  but displays a separate receptor for AI-2,  namely the two component sensor kinase LuxPQ [14]. Although homologs to LuxPQ have been found in other *Vibrio *species [15-18],  other bacterial species were reported to lack similar QS-2 related proteins with the exception of LuxS,  raising the prospect that this kind of receptor and signal transduction pathway is limited to the Vibrionales [19]. In *Salmonella typhimurium *other genes were shown to encode for a different complex that serves as receptor for AI-2 [20]. An ABC-type transporter named Lsr (Lux S-regulated) is responsible for the AI-2 uptake into the cell and was reported subsequently also in *Escherichia coli *[21]. AI-2 is phosphorylated inside the cell and is anticipated to interact with LsrR,  a protein that contributes to repress the *lsr*-operon and possibly acts as regulator of gene expression [20,22]. This mechanism is different from AI-2 detection in *V. harveyi*,  where just the signal but not the AI-2 molecule is transduced inside the cell. Thus alternative explanations to QS have been proposed whereby AI-2 may be released as a waste product and then reused subsequently as a metabolite [23,24] or,  as in the case of *Actinobacillus actinomycetemcomitans*,  that the molecule may be used as a borate scavenger by bacteria [25].

**Figure 1:**
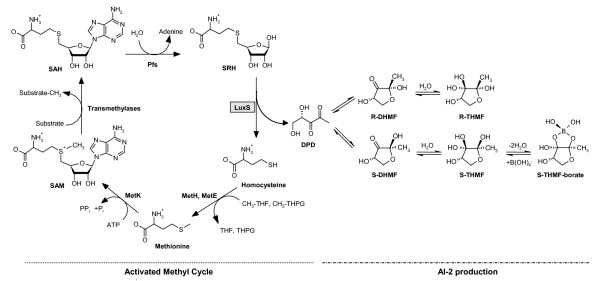
**Relation between the Activated Methyl Cycle (AMC) and AI-2 production in bacteria**. The AMC is responsible for the generation of the major methyl donor in the cell, S-adenosyl-L-methionine (SAM) and the recycling of methionine by detoxification of S-adenosyl-L-homocysteine (SAH). LuxS takes part in this cycle by salvaging the homocysteine moiety from the cycle intermediate S-ribosyl-homocysteine (SRH). As a by-product of this reaction the direct AI-2 precursor 4,5-dihydroxy-2,3-pentadione (DPD) is formed. DPD undergoes further reactions to form distinct biologically active signal molecules generically termed AI-2. (2S,4S)-2-methyl-2,3,3,4-tetrahydroxytetrahydrofuran-borate (*S*-THMF-borate), the AI-2 signal of Vibrionales, is produced without the help on any known enzyme in the presence of boric acid (lower pathway), while in other bacteria (e.g., *S. typhimurium*) DPD rearranges spontaneously to form (2R,4S)-2-methyl-2,3,3,4-tetrahydroxytetrahydrofuran (*R*-THMF) as AI-2 signal (upper pathway). CH_3_-THPG: *N*^*5*^-methyltetrahydropteroryl glutamate, CH_3_-THF: *N*^*5*^-methyltetrahydrofolate.

There are now several studies that describe the central role of LuxS in *E. coli *as crucial for the transition to pathogenic existence inside the host [26,27] by controlling virulence determinants such as motility [28],  biofilm formation [29] or gene expression [30]. AI-2 dependent behavior was consequently claimed also in other Gram-negative and Gram-positive bacteria (Tables 1,  2,  3 and additional file 1, Table 1s),  but many reports are simply based on the presence of the *luxS *gene,  mutant analysis and/or additional phenotypic tests such as the use of AI-2 reporter strain *V. harveyi *BB170 [27]. Rarely have investigations examined the existence of potential AI-2 receptors or assessed their functionality by proving AI-2 depletion of the media by the wildtype or by performing a chemical complementation of *luxS *mutants using pure AI-2 signal or AI-2 conditioned supernatants [31,32]. This is essential since it is now well recognized that LuxS displays also a primary role as metabolic enzyme in the activated methyl cycle (AMC) [11], which is responsible for the generation of the cell major methyl donor S-adenosyl-L-methionine (SAM) and the recycling of methionine by detoxification of S-adenosyl-L-homocysteine (SAH). LuxS takes part in this cycle by salvaging the homocysteine moiety from the cycle intermediate *S*-ribosyl-homocysteine (SRH). As a by-product of this reaction, the direct AI-2 precursor (S)-4,5-dihydroxy-2,3-pentanedione (DPD) is formed. In *V. harveyi *DPD cyclizes and is spontaneously converted to furanosyl borate diester by the addition of borate [33],  while *S. typhimurium *produces and recognizes a chemically distinct form of AI-2 [34] (Fig. 1). Given this dual nature of LuxS the sole use of the commonly exploited mutational approach is inappropriate,  since a simple genetic complementation using the intact gene on a plasmid will also repair the induced metabolic unbalance as well and yield in any case a restored phenotype.

**Table 1:** A literature survey of the relationship between the presence of the *luxS *gene and QS-2 dependent behavior in bacterial species carrying either a *luxP*- or a *lsrB*-gene homolog in their genome

	**Talk**^**a**^	**Listen**^**a**^		**Answer**^**b**^		
**Strain**	***luxS***	***luxP***	***lsrB***	**AI-2 related phenotype**	**Chemical compl.**^**c**^	**References**
*Actinobacillus actinomycetemcomitans*	+	-	+	yes	+/SN	[45]
				yes	nd	[70]
				yes	+/AI-2	[68]
				yes	+/AI-2	[25,52]
*Bacillus anthracis*	+	-	+	yes	nd	[71]
*Bacillus cereus*	+	-	+	yes	+/AI-2	[72]
*Bacillus thuringensis*	+	-	+	nd	nd	-
*Desulfovibrio desulfuricans*	-	(+)	-	nd	nd	This work
*Escherichia coli*	+	-	+	yes	+/SN	[73]
				yes	+/SN	[30]
				yes	+/SN	[21]
				Q	nd	[74]
*Haemophilus somnus*	+	-	+	H	nd	[75]
*Klebsiella pneumoniae*	+	-	+	H	nd	[76]
*Pasteurella multocida*	+	-	+	H	nd	[77]
*Photorhabdus luminescens*	+	-	+	yes	nd	[56]
				yes	+/AI-2	[78]
*Rhodobacter capsulatus*	-	-	+	nd	nd	This work
*Rhodobacter sphaeroides 2.4.1*	-	-	+	nd	nd	This work
*Salmonella enterica*	+	-	+	yes	nd	[79]
*Salmonella typhimurium*	+	-	+	yes	+/AI-2	[20]
				yes	+/AI-2	[22]
*Marinomonas *sp. MED121	-	(+)	-	nd	nd	This work
*Neptuniibacter caesariensis*	-	(+)	-	nd	nd	This work
*Sinorhizobium meliloti*	-	-	+	nd	nd	This work
*Shigella dysenteriae*	+	-	+	nd	nd	-
*Shigella flexneri*	+	-	+	yes	+/SN	[80]
*Vibrio anguillarum*	+	+	-	yes	nd	[81]
*Vibrio cholerae*	+	+	-	yes	+/SN	[82]
*Vibrio harveyi*	+	+	-	yes	+/SN	[83]
*Vibrio fischeri*	+	+	-	yes	+/SN	[84]
*Vibrio parahaemolyticus*	+	+	-	yes	+/SN	[85]
*Vibrio vulnificus*	+	+	-	yes	+/SN	[38]
*Yersinia pestis*	+	-	+	nd	nd	[86]
				nd	nd	[87]

A more detailed description of the findings of the papers cited herein is available as additional file 1 (Table 1s).

^a ^+: positive (>50% protein identity); (+): positive (<50% protein identity); -: negative (<30% protein identity);

^b ^H: hypothesized; nd: not determined; Q: questioned;

^c ^+: successful complementation; -: unsuccessful complementation; nd: not determined; AI-2: complementation with pure autoinducer; DPD: complementation with precursor; SN: complementation with co-culture or conditioned supernatants of *luxS*-positive strain.

**Table 2:** A literature survey of the relationship between the presence of the *luxS *gene and QS-2 dependent behavior in bacterial species without a *luxP*- or a *lsrB*-gene homolog in their genome for which a metabolic role was hypothesized in at least one work.

	**Talk**^**a**^	**Listen**^**a**^		**Answer**^**b**^		
**Strain**	***luxS***	***luxP***	***lsrB***	**AI-2 related phenotype**	**Chemical compl.**^**c**^	**References**
*Actinobacillus pleuropneumoniae*	+	-	-	none	-/SN	[88]
*Borrelia burgdorferi*	+	-	-	C	-/SN	[89]
				C	nd	[90]
				C	+/DPD	[91]
				none	nd	[92]
				none	nd	[41]
*Clostridium difficile*	+	-	-	H	-/AI-2	[93]
				C	+/SN	[94]
*Erwinia amylovora*	+	-	-	none	-/SN	[95]
				C	nd	[96]
*Helicobacter pylori*	+	-	-	C	nd	[97]
				C	nd	[98]
				none	nd	[99]
				C	nd	[100]
				none	nd	[101]
*Lactobacillus rhamnosus*	+	-	- ^d^	none	-/DPD	[102]
*Listeria monocytogenes*	+	-	-	none	-/SN	[103]
				none	-/AI-2	[104]
*Neisseria meningiditis*	+	-	-	C	nd	[105]
				none	-/AI-2	[106]
*Proteus mirabilis*	+	-	-	none	nd	[107]
*Serratia plymuthica*	+	-	-	none	nd	[108]
*Staphylococcus aureus*	+	-	-	none	-/SN	[109]
*Streptococcus mutans*	+	-	-	C	+/AI-2	[50]
				C	+/SN	[49]
				C	nd	[110]
				none	nd	[111]
*Streptococcus pyogenes*	+	-	-	none	-/SN	[112]
				C	nd	[113]
				C	nd	[114]
*Vibrio angustum*	+	-	-	none	nd	[39]

A more detailed description of the findings of the papers cited herein is available as additional file 1 (Table 1s).

^a ^+: positive (>50% protein identity); (+): positive (<50% protein identity); -: negative (<30% protein identity);

^b ^C: claimed; H: hypothesized;

^c ^+: successful complementation; -: unsuccessful complementation; nd: not determined; AI-2: complementation with pure autoinducer; DPD: complementation with precursor; SN: complementation with co-culture or conditioned supernatants of *luxS*-positive strain;

^d ^Hypothesized on the basis of the genomes of 47 fully sequenced Lactobacillales (all *lsrB*-negative).

**Table 3:** A literature survey of the relationship between the presence of the *luxS *gene and QS-2 dependent behavior in bacterial species without a *luxP*- or a *lsrB*-gene homolog in their genome for which the presence of a QS-2 system was claimed

	**Talk**^**a**^	**Listen**^**a**^		**Answer**^**b**^		
**Strain**	***luxS***	***luxP***	***lsrB***	**AI-2 related phenotype**	**Chemical compl.**^**c**^	**References**
*Actinomyces naeslundii*	+	-	-	C	+/DPD	[115]
*Bacillus subtilis*	+	-	-	C	+/SN	[116]
*Bifidobacterium adolescentis*	+	-	-	nd	nd	This work
*Bifidobacterium longum*	+	-	-	nd	nd	[11]
*Campylobacter jejuni*	+	-	-	C	nd	[117]
				C	nd	[118]
*Clostridium perfringens*	+	-	-	C	+/SN	[119]
*Enterococcus faecalis*	+	-	-	nd	nd	[120]
*Pectobacterium carotovora*	+	-	-	C	nd	[121]
				C	nd	[122]
*Escherichia blattae*	+	-	nd	nd	nd	[43]
*Photobacterium profundum*	+	-	-	nd	nd	[123]
*Porphyromonas gingivalis*	+	-	-	C	nd	[124]
				C	nd	[125]
				C	nd	[46]
				C	+/SN	[126]
				C	+/SN	[127]
*Pseudomonas aeruginosa*	-	-	-	C	+/SN	[128]
*Serratia marcescens*	+	- ^d^	- ^d^	C	+/SN	[129]
*Shewanella *spp.	+	-	-	C	nd	[130]
*Staphyoycoccus epidermis*	+	-	-	C	+/SN	[131]
*Streptococcus anginosus*	+	-	- ^e^	C	+/DPD	[132]
				C	Interference	[133]
*Streptococcus gordonii*	+	-	- ^e^	C	nd	[46]
*Streptococcus pneumoniae*	+	-	-	C	nd	[134]

A more detailed description of the findings of the papers cited herein is available as additional file 1 (Table 1s).

^a ^+: positive (>50% protein identity); (+): positive (<50% protein identity); -: negative (<30% protein identity); nd: not determined;

^b ^nd: not determined; C: claimed;

^c ^+: successful complementation; -: unsuccessful complementation; nd: not determined; AI-2: complementation with pure autoinducer; DPD: complementation with precursor; SN: complementation with co-culture or conditioned supernatants of *luxS*-positive strain;

^d ^Hypothesized based on *Serratia proteomaculans *sequence;

^e ^Hypothesized on the basis of more than 10 known *Streptococcus *complete genomes belonging to the species *S. pneumoniae*, *S. mutans, S. pyogenes*, *S. agalactiae*, *S. suis*, *S. equis *and *S. zooepidemicus*.

In order to assess whether the role of LuxS in well-described bacterial species is effectively related to AI-2 mediated autoinduction (or AI-2 uptake),  we performed a database analysis for established AI-2 receptors in fully-sequenced genomes and sequence databases,  and compared the outcome with published results about QS-2 in these bacteria. In this way we were able to corroborate many reports where the role of LuxS in QS-2 was proven experimentally (accordingly an AI-2 receptor was found using our *in silico *screening) or to confirm results of other works where no effect related to autoinduction could be demonstrated (in this case no known receptor could be discovered in the sequence). On the other hand,  in those cases when an AI-2 mediated mechanism was claimed,  but no known receptor was found in the corresponding bacterial sequence,  novel or alternative receptors must be postulated or,  without a successful chemical complementation of the *luxS *mutant,  a sheer metabolic role must be acknowledged.

With the aim of understanding the evolutionary relationships among LuxS,  the presence of AI-2 receptors and the functionality of the QS-2 system,  a phylogenetic analysis was performed to compare the evolution of the *luxS *gene with the presence and evolution of the genes coding for known AI-2 receptors,  transporters and regulators. Since known AI-2 receptors genes are (with very few exceptions) restricted to *luxS*-positive bacteria and have only partially coevolved with the AI-2 synthase,  it can be hypothesized that the QS-2 function was acquired and developed as a new trait by some bacteria by building upon the preexistent metabolic function of LuxS.

## Methods

### Search for known AI-2 receptors in complete genomes

Complete genome sequences of bacteria known to have a *luxS *gene were analyzed for the presence of AI-2 receptor sequences using the blastx and the tblastx programs with a cut-off E-value of 1E-80 by applying a reciprocal best hit strategy,  which allows to distinguish between orthologous and paralogous genes [35]. As starting queries the *luxP *gene of *V. harveyi *strain BB7 (accession number U07069) and the *lsrB *gene of *E. coli *strain K12 (accession number NC_000913,  locus tag b1516) were used. The hits were manually checked to confirm that they had the same functional annotation as the query and re-submitted as novel queries until no new relevant positive match was found. In this second phase also *luxS*-negative strains were included in the analysis by performing a general blast search across the entire NCBI database . In bacteria found to carry a gene coding for an AI-2 receptor the presence of a complete QS-2 system was corroborated by verifying the existence of other genes associated to the respective AI-2 transduction pathway. Bacterial species were scored as positive for QS-2 receptors only when in addition to *luxP*,  homologs of the *luxQ *(inner membrane sensor kinase)*,  luxU *(two-component phosphorelay protein) and *luxO *(response regulator) genes which encode the full transduction pathway [12,13] were also found. Similarly,  positive results obtained with *lsrB *were confirmed by verifying the presence of genes constituting a complete *lsr*-operon (*lsrBDCARKG*). These include *lsrA *(encoding the ATPase that provides energy for AI-2 transport),  *lsrC *and *lsrD *(whose products form the heterodimeric membrane channel for the uptake of AI-2),  *lsrR *(encoding the repressor of the *lsr*-operon active in the absence of phospho-AI-2),  and *lsrK *(encoding the AI-2 kinase) [22].

Our investigation was extended to incomplete sequenced bacteria known to carry a *luxS *gene or for which a function of QS-2 was documented in the literature through an NCBI database search with relevant terms such as "*luxS*",  "*lsrB*",  "*luxP*",  "AI-2" or "quorum sensing". Discovered pertinent sequences were used in the analysis.

A literature search for published studies on the effect of type-2 quorum sensing in the organisms carrying the *luxS *gene was performed using the NCBI database and the papers were critically read,  with particular attention to those key experiments which are essential to ascertain the presence of a QS-2,  such as the identification of AI-2 receptors,  chemical complementation of the *luxS *mutant or detection of AI-2 depletion in the culture medium [31]. In cases where these tests were omitted the possibility that a metabolic dysfunction in the AMC may have been misinterpreted as disruption of type-2 quorum sensing was proposed.

### Phylogenetic analysis

Phylogenetic analysis was performed in order to understand how AI-2 receptors may have (co-)evolved in different bacteria with respect to the presence of *luxS*. Phylogenetic trees were generated on the basis of complete *luxS*- and *lrsB*-sequences without choosing any outgroup. Housekeeping gene *rpoB*,  encoding the RNA polymerase β-subunit,  was used as reference. Retrieved DNA sequences were aligned with ClustalW [36]. Sites presenting alignment gaps were excluded from analysis. The Molecular Evolutionary Genetics Analysis (MEGA) program version 4.0 [37] was used to calculate evolutionary distances and to infer trees based on the neighbor-joining (NJ) method using the JC formula. Nodal robustness of the inferred trees was assessed by 1000 bootstrap replicates.

### Genome resources and NCBI accession numbers

Complete genomes and key *luxS and lsrB *sequences were used.

#### Complete genomes

*Actinobacillus actinomycetemcomitans *(genome project for strain HK1651,  ,  *Actinobacillus pleuropneumoniae *(NZ_AACK01000006 and NC_009053),  *Actinomyces naeslundii *(genome project for strain MG1,  ,  *Agrobacterium tumefaciens *C58 (NC_003062-65),  *Bacillus anthracis *str. Sterne (NC_005945),  *Bacillus cereus *ATCC 14579 (NC_004722),  *Bacillus cereus *subsp. *cytotoxis *NVH 391-98 (NC_009674),  *Bacillus subtilis *subsp. *subtilis *str. 168 (NC_000964),  *Bacillus thuringensis *serovar *israelensis *ATCC 35646 (NZ_AAJM00000000),  *Bacillus thuringiensis *serovar *konkukian *str. 97-27 (NC_005957),  *Bacillus weihenstephanensis *KBAB4 (NC_010184),  *Bifidobacterium adolescentis *(NC_008618),  *Bifidobacterium longum *(NZ_AABM02000006), *Borrelia burgdorferi *(NC_001318),  *Campylobacter jejuni *(NC_003912), *Clostridium acetobutylicum *(NC_003030), *Clostridium difficile *(NC_009089), *Clostridium perfringens *(NC_008262)*,  Desulfovibrio desulfuricans *(NC_007519),  *Desulfuromonas acetoxidans *(NZ_AAEW00000000),  *Erwinia amylovora *(genome project for strain Ea273 ,  *Erwinia carotovora *subsp. *atroseptica *SCRI1043 (NC_004547), *Escherichia coli *(NC_000913),  *Haemophilus influenzae *(NZ_AADO01000002),  *Haemophilus somnus *(NZ_AACJ01000020),  *Helicobacter pylori *(NC_008086),  *Klebsiella pneumoniae *(genome project for strain MGH 78578,  ,  *Listeria monocytogenes *(NC_002973), *Mannheimia succiniciproducens *(NC_006300),  *Neisseria meningiditis *(NC_008767),  *Pasteurella multocida *(NC_002663),  *Photobacterium profundum *(NC_006370),  *Photorhabdus luminescens *(NC_005126),  *Rhodobacter sphaeroides *2.4.1 (NC_007493-94),  *Rhodobacter sphaeroides *ATCC 17025 (NC_009428),  *Rhodobacter sphaeroides *ATCC 17029 (NC_009049-50),  *Salmonella bongori *(genome project for strain 12419 ATCC 43975,  ,  *Salmonella enterica *(NC_006905),  *Salmonella typhimurium *(NC_003197), *Serratia marcescens *(genome project for strain Db11 ),  *Shewanella oneidensis *MC_1 (NC_004347),  *Shighella boydii *(NZ_AAKA01000004),  *Shighella dysenteriae *(NZ_AAMJ01000009),  *Shighella flexneri *(NC_004337),  *Shighella sonnei *(NC_007384),  *Sinorrhizobium meliloti *(NC_003078),  *Sodalis glossinidius *(NC_007712),  *Staphylococcus aureus *(NC_003923),  *Streptococcus pyogenes *(NC_003485),  *Vibrio angustum *(NZ_AAOJ00000000),  *Vibrio cholerae *(NC_002505),  *Vibrio fischeri *(NC_006840), *Vibrio harveyi *(NC_009783-NC_009784),  *Vibrio vulnificus *(NC_004459), *Xenorhabdus *sp. (genome project for *X. nematophila *ATCC 19061 and *X. bovienii*,  ,  *Yersinia bercovieri *(NZ_AALC00000000),  *Yersinia enterocolitica *(NC_008800), *Yersinia frederiksenii *(NZ_AALE01000042),  *Yersinia intermedia *(NZ_AALF01000074),  *Yersinia mollaretii *(NZ_AALD01000064), *Yersinia pestis *(NC_004088), *Yersinia pseudotubercolosis *(NC_006155).

#### *luxS *gene sequences

*Erwinia billingiae *(DQ977724),  *Erwinia tasmaniensis *(AM117930),  *Vibrio anguillarum *(DQ466077).

#### *lsrB *gene sequences

*Rhodobacter capsulatus *(U57682).

## Results

### Presence of *luxP*-type receptor genes

**Figure 2:**
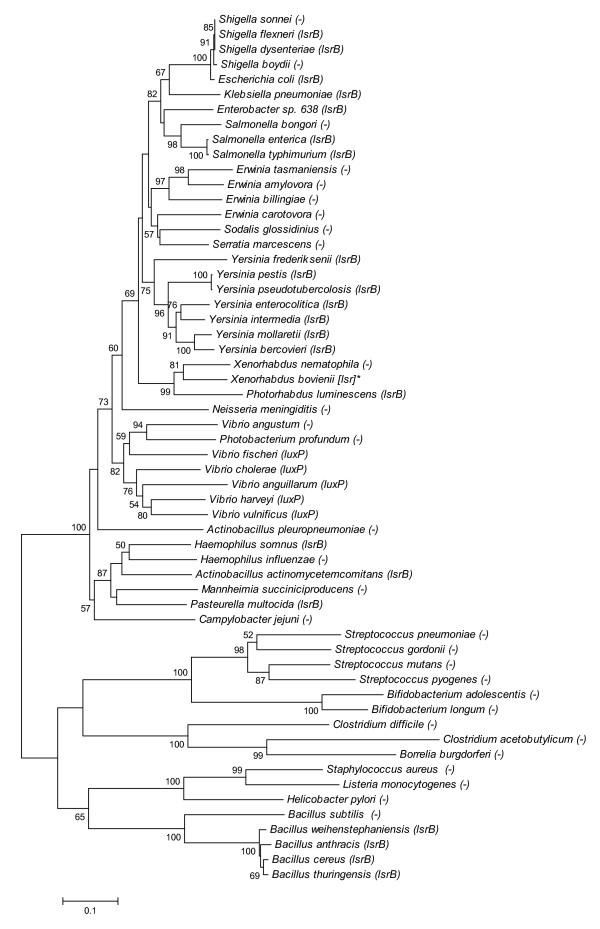
**Phylogenetic relationships among Gram-positive and Gram-negative bacteria on the basis of complete *luxS *sequences and presence of established AI-2 receptors in the respective genomes**. The distance tree was generated by the NJ method with the JC formula, without choosing any outgroup. Nodal supports were assessed by 1000 bootstrap replicates. Only bootstrap values greater than 50% are shown. The scale bar represents the number of substitutions per site. The presence of recognized AI-2 receptor genes (*luxP*, *lsrB*) is indicated between parentheses. The asterisk shows the truncated Lsr-receptor complex of *X. bovienii*. With the exception of the *E. billingiae*, which was produced in this work (accession number DQ977724), all *luxS *sequences were retrieved at the NCBI database or in published genome projects (see Methods for accession numbers).

The *luxP *gene of *V. harveyi *strain BB7 was used as a starting query for a search of LuxPQ-type receptors in bacterial genomes that was successful only for members of the orders of the Vibrionales and,  to a much lesser extent,  of the Oceanospirillales. This kind of receptor,  along with the effector kinase LuxQ,  was already known in several species of the genus *Vibrio*,  i.e., *V. anguillarum*,  *V. cholerae*,  *V. fischeri*,  *V. harveyi *and *V. parahaemolyticus *(see Table 1 and citations therein). In *Vibrio *species for which a response to AI-2 system is documented,  but in which no receptor was yet described,  i.e.,  *V. vulnificus *[38] and *V. alginolyticus *[39],  we were able to retrieve the *luxPQ *genes by searching the relevant gene sequences in the corresponding published genomes. Conversely,  the LuxPQ-receptor was absent from the genome of *V. angustum*,  which does not respond to the AI-2 signal [39],  even though the *luxS *gene coding for S-ribosylhomocysteinase is present. Besides that,  we managed to identify the LuxPQ receptor complex also in other representatives of the genus for which no study about QS-2 related phenotype has been performed to date,  i.e.,  *V. splendidus*,  *Vibrio *sp. Ex25 and *Vibrio *sp. MED222,  but not in closely related *Photobacterium profundum *SS9 (Fig. 2 and Table 3).

A LuxP homolog was found also in the genomes of some Oceanospirillales,  albeit among the bacteria of this order no *luxS *gene has been sequenced or found to date. The similarity of these proteins to the LuxP-receptor present in *V. harveyi *was only moderate: 41.2% identity and 71.7% similarity for the *Neptuniibacter caesariensis *MED92 periplasmic protein (locus tag: MED92_04774) which carries a region similar to the RbsB-like ABC-type sugar transport system receptor locus and 24.5% identity and 61.8% similarity for the *Marinomonas *sp. MED121 LuxP protein precursor (locus tag: MED121_11334). However,  both results withstood the reciprocal best hit strategy test and yielded a LuxP protein of *Vibrio *as best match in a blastx search. Furthermore,  in *Neptuniibacter caesariensis *MED92 and *Marinomonas *sp. MED121 the genes encoding these putative LuxP homologs are arranged in an operon with presumed transcriptional regulators that likewise contain PAS domains (locus tags MED92_04779 and MED121_11339 respectively),  but share otherwise little homology with each other. In *Marinomonas *sp. MED121,  the transcriptional regulator showed moderate homology with LuxQ of *V. cholerae *and contains also AAA-type ATPase and DNA-binding domains and is followed by the *pfs *gene coding for a 5'-methylthioadenosine nucleosidase (locus tag: MED121_11344). A similar protein (locus tag Dace_1280) in *Desulfuromonas acetoxidans *(a δ-Proteobacterium) showed an equivalent identity to LuxP of *V. harveyi *(36.2%) and was analogously arranged in an operon with a putative transcriptional regulator containing PAS domains (locus tag Dace_1279), while in *Desulfovibrio desulfuricans *(also a δ-Proteobacterium) the matching protein (locus tag Dde_3311, identity 41.0%) also carries a putative receptor locus but the corresponding gene was not associated with either a *luxQ *homolog (or a similar transcriptional regulator) or the other usual genes of an ABC-transporter, indicating the probable absence of a signal transduction pathway toward the inner of the cell. However, also Dace_1280 and Dde_3311 pointed toward LuxP in the reciprocal best hit test. As for Oceanospirillales, both *D. acetoxidans *and *D. desulfuricans *(both δ-Proteobacteria) were found to lack the *luxS *gene. All Vibrionales carrying the *luxPQ *genes also carried open reading frames which encoded proteins homologous to the two component, σ^54^-specific, transcriptional regulator LuxUO found in *V. harveyi *(accession number L26221). In Oceanospirillales a similar situation was discovered, but both *luxP *and *luxO *were flanked by other transcriptional regulator genes which displayed a less important resemblance with *luxQ *and *luxU *respectively. In all strains investigated the LuxO protein displayed the characteristic 8-AA glycine-rich consensus (GESGTGKE) which is needed for interaction with alternative σ^54 ^(data not shown) [40]. Interestingly, in none of the strains were the genes encoding the single components of the QS-2 mechanism (*luxS*, *luxPQ *and *luxUO*) found in proximal positions on the genome (data not shown), thus they were probably acquired and/or developed independently.

Notably the tblastx search using *luxP *gene of *V. harveyi *strain BB7 resulted in local hits with the *lsrB *gene of Enterobacteriales (E-values > 1E-10). Similarity is limited to a small RbsB-like portion on the C-terminus of the LuxP protein, but is most probably indicative of the substrate (AI-2) binding domain of both receptors. Generally, the use of *luxQ *gene of *V. harveyi *strain BB7 as starting query yielded multiple partial positive matches in almost every strain tested, but these were limited to the cytoplasmic transduction domain of other sensor kinases and were not directly related to any known quorum sensing related gene (data not shown).

### Presence of *lsrB*-type receptor genes

Our screening of completed genome projects for *lsrB*, the gene belonging to the *lsr*-operon encoding the periplasmic AI-2 binding component of the ABC-type sugar transport apparatus, revealed that this type of receptor is generally more widely spread than the LuxPQ-system. We found that *lsrB *is present both in Gram-negative and in Gram-positive bacteria, albeit its distribution was not homogeneous in and among the bacterial orders considered (Fig. 2 and Tables 1, 2, 3). An open reading frame coding for LsrB was found, for Gram-negative γ-Proteobacteria, essentially only in part of Enterobacteriales (i.e., in *E. coli*, *Photorhabdus luminescens*, *Klebsiella pneumoniae*, *Yersinia *spp., in *Shigella dysenteriae *and *Shigella flexneri*, and *Salmonella *spp. with the exception of *S. bongori*, but not in *S. marcescens*, *S. glossinidius *and in *Erwinia *spp.) and of Pasteurellales (i.e., in *Haemophilus somnus*, *Pasteurella multocida *and *Actinobacillus actimycetemcomitans*, but not in related species such as *Haemophilus influenzae *or *Actinobacillus pleuropneumoniae*). No evidence for *lsrB *was found in Vibrionales and in the *luxS*-negative families of Pseudomonadaceae and Xanthomonadaceae. No *lsrB *homolog was located either in the *luxS*-positive class of ε-Proteobacteria (*Helicobacter*, *Campylobacter*) or in Spirochaetales, in which the presence of *luxS *was reported to date only in *Borrelia *spp. [41].

For Gram-negative α-Proteobacteria, *lsrB *was found only on the pSymB-plasmid of *Sinorhizobium meliloti *and in three *Rhodobacter *species, i.e. *R. capsulatus *(contig hit, position on the genome unspecified) and on the high variable chromosome-2 of *R. sphaeroides *2.4.1 and *R. sphaeroides *ATCC 17029, but was absent from *R. sphaeroides *ATCC 17026. These finding are of special interest since α-Proteobacteria are lacking the *luxS *gene coding for the S-ribosylhomocysteinase/AI-2 synthase.

In Gram-positive bacteria, *lsrB *was located solely in bacteria belonging to the *Bacillus cereus *group. *B. anthracis*, *B. thuringensis*, *B. weihenstephanensis *and *B. cereus *were found to carry the *lsr*-operon (see additional file 2, Figure 1s). However, in *B. weihenstephanensis *KBAB4 both *lsrB *and *lsrD *show frameshifts and are listed as pseudogenes, in a similar way to *lsrB *of *B. thuringensis *serovar *konkukian *97-27 and to the associated ATPase-gene *lsrA *of non-pathogenic *B. cereus *ATCC 14579, which is apparently truncated. In *B. thuringensis *serovar *israeliensis *ATCC 35646 a pseudogene with very high homology to part of the *lsrB*-receptor gene is found well separated from the rest of the operon (*lsrDCARK*) suggesting that in the latter strain the ABC-transporter was inactivated by a rearrangement of the genome. The *lsrB *gene could not be identified in closely related *Bacillus cereus *ssp. *cytotoxis *NHV391-88 confirming a significant degree of divergence from typical *B. cereus *[42], which is corroborated also by our *luxS*-based phylogeny. More distant *Bacillus *species, including *B. subtilis*, were all found to be *lsrB*-negative (see additional file 2, Figure 1s) suggesting that in Gram-positive bacteria the Lsr-transporter was acquired or developed by an ancestor of the *Bacillus cereus *group. No *lsrB *homolog was found in the Actinobacteria *Bifidobacterium longum *[11] and *Bifidobacterium adolescentis *which are so far the only bacteria besides *Escherichia blattae *[43], to possess both known SAH-detoxification pathways (SAH hydrolase and Pfs/LuxS).

All strains which yielded a positive match for *lsrB *were further verified by demonstrating the presence of other genes (*lsrDCARKG*) encoding the Lsr-complex. However, in none of the positive matches the *luxS *gene and the *lsr *operon were found to lie in proximal regions of the genome, suggesting that (as for *luxPQ *and *luxUO *in Vibrionales) they were probably acquired and/or developed independently. On the other hand, in Enterobacteriaceae *luxS *is frequently found in association with another gene involved in sulfur metabolism such as *gshA*, coding for the γ-glutamate cysteine ligase (data not shown).

An ambiguous positive match to LsrB (67.1% similarity, 31.4% identity) was obtained for substrate binding protein Atu3487 (accession number NC_003305) on the linear chromosome of *Agrobacterium tumefaciens *C58. However, both the sequence of the receptor and the organization of the flanking genes in the operon displayed high similarity with the rhamnose catabolism gene locus of *Rhizobium leguminosarum *bv. *trifolii *plasmid pRleW14-2c (accession number AF085687) showing that these genes are probably not involved in quorum sensing. Similarly, a positive match (23.8% similarity, 19.1% identity) in *Actinobacillus pleuropneumoniae *to an uncharacterized conserved protein (locus tag Aple02001327) was discarded after additional analysis yielded that the retrieved sequences showed more similarity to *E. coli xylF *gene, coding for the membrane receptor protein of the ABC-type xylose transport system.

### Phylogenetic analysis

In order to understand how QS-2 developed in the different bacterial taxa during evolution, a phylogenetic analysis was performed by comparing, for those species for which a QS-2 has been previously described, the presence of the established AI-2 receptor genes *luxP *and *lsrB *to the tree based on the *luxS*-gene sequences (Fig. 2). Our results confirmed the conclusions of a previous work [19] in which a few species appeared be misplaced on the *luxS *tree if compared to species phylogeny and thus to have acquired the latter gene by an horizontal gene transfer (HGT) event: these bacteria are β-proteobacterium *Neisseria meningiditis*, ε-proteobacterium *Helicobacter pylori*, the spirochete *Borrelia burgdorferi*, and actinobacteria *Bifidobacterium longum *and *Bifidobacterium adolescentis*. Interestingly, however, in none of these bacteria the gene sequences encoding the LuxP- or LsrB-receptors have been found, indicating that most probably only the metabolic function of LuxS, and not a complete QS-2 system, was transferred.

More generally, as detailed above, the occurrence of *luxP *was restricted to Vibrionales, but the gene was not universally present in every species of this order, missing for instance from *Vibrio angustum *or *Photobacterium profundum*. Similarly, the presence of the *lsrB*-gene was generally limited to certain species belonging to the Gram-negative bacterial orders of Enterobacteriales. Pasteurellales, and to the Gram-negative positive classes of Bacilli and Clostridia and was not a collective feature of all the members of each group. Most importantly, several species for which an AI-2 dependent behavior was claimed (Table 3) were found to be devoid of either receptor genes.

**Figure 3:**
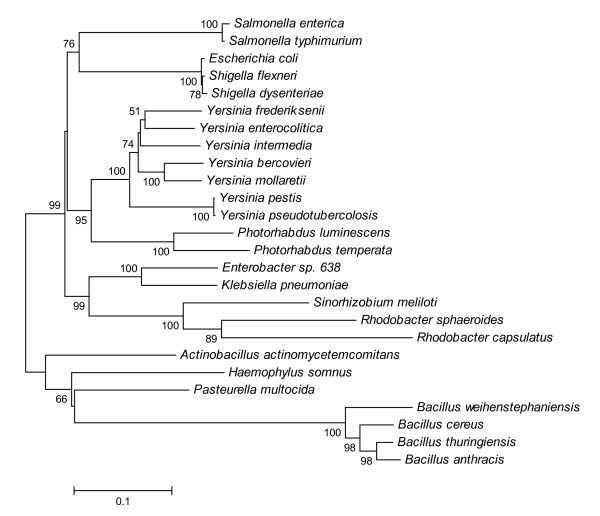
**Phylogenetic relationships on the basis of complete *lsrB***. The distance tree was generated by the NJ method with the JC formula, without choosing any outgroup. Nodal supports were assessed by 1000 bootstrap replicates. Only bootstrap values greater than 50% are shown. All *lsrB *sequences were retrieved at the NCBI database or in published genome projects (see Methods for accession numbers).

Analysis of *lsrB *tree (Fig 3) shows that this gene is well distinct from related ABC-receptor genes such as *rbsB *or *xylF*. This result is supported by similar analyses performed on the other genes coding for the ABC-receptor complexes (data not shown) and indicates that the Lsr receptor is ortholog to those ABC-transporters and did not arose by gene duplication from related sugar transporters in each single species or genus. The *lsrB*-phylogeny of *luxS*-negative species *R. sphaeroides*,*R. capsulatus *and *S. meliloti *reveals that the Lsr-receptor complex of these α-proteobacteria was probably horizontally acquired from unrelated enterobacterial species. The HGT hypothesis is further supported by the fact that in these species the genes coding for the AI-2 receptor are not on the main chromosome, but are found on dynamic genetic elements such as a smaller chromosome as in the case of *R. sphaeroides *2.4.1 or on a plasmid as in *S. meliloti *1021.

**Figure 4:**
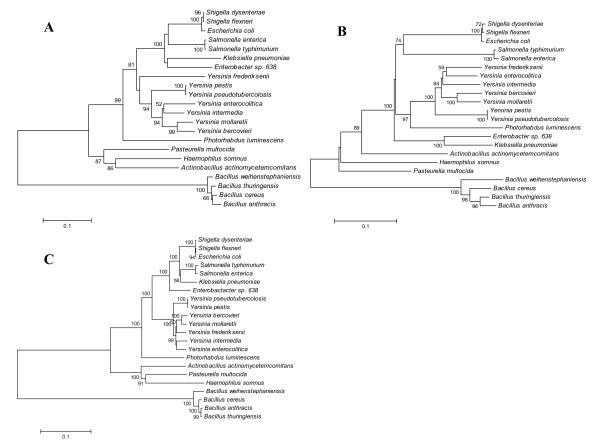
**Comparison of phylogenetic trees based on complete sequences of *luxS *(A), *lsrB *(B) and *rpoB *(C)**. All trees are restricted to strains which are both *luxS*- and *lsrB*-positve. The distance tree was generated by the NJ method with the JC formula, without choosing any outgroup. Nodal supports were assessed by 1000 bootstrap replicates. Only bootstrap values greater than 50% are shown. The scale bar represents the number of substitutions per site. All sequences were retrieved at the NCBI database or in published genome projects (see Methods for accession numbers).

Taking in consideration only *lsrB*-positive bacteria, we finally compared the topology of the phylogenetic trees based on *luxS*, *lsrB *and the housekeeping gene *rpoB*, encoding a subunit of the RNA polymerase and which is commonly used as a molecular marker for microbial evolution studies (Fig. 4). The *luxS*- and *rpoB*-trees displayed a branching pattern which was more similar to each other than to that of the *lsrB*-tree, however in the latter most of the differences were found at local level within single clades and (if present) they were not supported by high bootstrap values even when using more rigorous building methods such as minimum evolution or maximum parsimony (data not shown), indicating the *lsrB *may have undergone an HGT only in a limited number of cases. This result, together with the discrete distribution of *lsrB *among *luxS*-positive bacteria suggests that for *luxS- *and *lsrB*-positive bacteria the AI-2 receptor coevolved with the SAH-lyase/AI-2 synthase, but that for *luxS-*negative strains it was probably acquired by certain species (or their common ancestor) as adaptation to a specific niche.

## Discussion

### Distribution of LuxPQ sensor kinase

Our search across fully published genomes clearly shows that the presence of this kind of AI-2 receptors is primarily limited to a narrow group of bacteria. Corroborating the results of Sun and coworkers [19] no complete QS-2 receptor complex based on the LuxPQ sensor kinase and the related signal cascade was found in bacteria not belonging to the order of Vibrionales. Within the latter order the presence of *luxPQ *genes was not ubiquitous, but was found to be largely in accordance with the responsiveness to AI-2 stimulation and the functionality of the QS-2 system that can be deduced from the literature already published on this topic (Table 1). No alternative receptor for AI-2 has been described so far in bacteria belonging to the Vibrionales. Even if an experimental verification is still pending, we predict therefore that other sequenced *Vibrio *species (i.e., *V. splendidus*, *Vibrio *sp. Ex25 and *Vibrio *sp. MED222) that carry both LuxS, LuxPQ and LuxUO will most probably have a functional QS-2 system, while *Photobacterium profundum *SS9 will not.

LuxP homologs were also found in *luxS-negative Neptuniibacter caesariensis *MED92, *Marinomonas *sp. MED121 and *D. acetoxidans *DSM684. Since previous evidence suggests that AI-2 may also be involved in interspecies gene regulation [44,45] and mixed-species biofilm formation [46], it is therefore conceivable that some bacteria may be able to recognize the AI-2 signal even though they are unable to produce it. The evolutionary benefit would be the opportunity to sense the presence and abundance of AI-2 producers sharing the same niche. Both *Neptuniibacter *and *Marinomonas *are marine bacteria that share the same environment [47] or are associated with the same host as AI-2 producer *Vibrio *strains [48]. However, given the less important similarity to the receptor found in Vibrionales and the different sensor kinase associated this hypothesis has in any case to be proven experimentally. In the case of *D. desulfuricans*, in which the *luxP *homolog is not physically associated to any gene indicating a signal transduction toward the inner of the cell, the prospect of an AI-2 related QS seems less realistic.

### Distribution of Lsr-receptor complex

Although in recent years the activity of QS-2 was repeatedly hypothesized on the basis of the presence and functionality of the *luxS *gene in several bacterial species not belonging to the order of Vibrionales, few efforts have been made to verify the presence of a cognate AI-2 receptor, either experimentally by demonstrating successful chemical complementation of *luxS *mutant (using AI-2 containing supernatants) or genetically by showing the presence of the associated genes in the corresponding bacterial genomes. The only other clearly established AI-2 receptor, the Lsr ABC-transporter was identified so far only in *S. typhimurium *[20] and *E. coli *[21]. For this reason we examined the genomes of completely sequenced bacteria with a reported quorum sensing activity ascribed to AI-2 and searched for the presence of DNA-sequences coding for receptor protein LsrB, the extracellular substrate-binding component of the Lsr-complex.

The outcome of our investigation was at first glance surprising, since a positive match was found only in part of Enterobacteriales, Pasteurellales and Bacillales (Fig. 2). However, a more detailed analysis of the results showed that these were largely congruent with the ability to complement the *luxS *mutation by the means of chemically pure AI-2 signal or AI-2 containing supernatants. The *lsrB *gene was retrieved exclusively in the genome of bacteria for which, provided that it was performed in the corresponding study, a chemical complementation yielded a successful result (Table 1). Conversely, our search for *lsrB *did not yield positive results in any of the species in which exogenous AI-2 was unable to restore the original wild-type phenotype in *luxS *mutants (Table 2). These results strongly corroborate the hypothesis that LsrB functions as receptor for AI-2 and the fact that in many bacteria the role of LuxS is restricted to the AMC.

In a few cases we were unable to identify either the Lsr-receptor complex or the LuxPQ sensor kinase in the genome of the corresponding bacteria even if a successful chemical complementation of the *luxS *mutant using AI-2 containing fluids was claimed in the literature (Tables 2 and 3), although it must be stressed that for some bacterial species at least one work came to contrasting conclusions and asserted a mere metabolic role for LuxS (Table 2). Strain-specific distribution or functionality of *lsr*-transporters are in principle possible explanations for this result. Although the *lsr*-operon seems to be ubiquitous in strains belonging to the *Bacillus cereus *group, in some non-pathogenic strains (e.g, *B. cereus *ATCC 14579) or insect-associated species like *B. thuringiensis *the sequences of the *lsr*-genes revealed frameshifts or rearrangements that will most likely lead to the production curtailed proteins and non-functional AI-2 receptors. Since in none of these strains the *lsr*-operon was completely absent from the genome, this may be the result of gene deterioration as a consequence of reduced selective pressure in those strains not tightly associated with warm-blooded animals.

### Alternative receptors

Another option to explain successful chemical complementation of *luxS*-mutants in bacteria lacking both LuxPQ or the Lsr-receptor complex is to postulate the presence of alternative receptors for AI-2. Yoshida and coworkers [49] were able to complement the defect in biofilm formation of a *Streptococcus mutans luxS *mutant by culture media of other Streptococci or other *luxS*-positive bacteria which are normally associated with *S. mutans *in the human oral cavity such as *Porphyromonas gingivalis *and *A. actinomycetemcomitans*, while in a more recent study 59 genes responsive to AI-2 were identified in the same strain by global transcriptome analysis [50]. In the latter study, the product of one of the strong AI-2 upregulated genes (SMU.408) was hypothesized to act as an uptake mechanism for the autoinducer molecule. The corresponding protein is a xanthine/uracil/vitamin C permease (COG2252) and belongs to the major facilitator superfamily (MFS) transporters. Homologs to this putative receptor are present in all *Streptococcus *species for which a QS-2 related phenotype was described, but also in many other species, including many for which a conventional receptor for AI-2 is already known, such as *V. vulnificus*, *B. anthracis *or *P. multocida *(complete data not shown).

A role as an alternative AI-2 receptor was also suggested for the ribose ABC-transporter encoded by the *rbs*-operon. Stevenson and coworkers speculated that RbsB may serve as the AI-2 receptor in *Borrelia burgdorferi *[51], while in *Actinobacillus actionmycetemcomitans *both the LsrB and RbsB proteins were shown to interact with AI-2 molecule [52]. The AI-2 signal showed differential affinity to two receptors depending if found as THMF (like in *S. typhimurium*) or as borate diester (like in *V. harveyi*) (Fig. 1) leading to the hypothesis that the two receptors may be used together by the bacteria to scavange borate from the environment [25]. The distribution of the ribose ABC-transporter is almost ubiquitous among most bacterial species regardless if the *luxS*- and *lsrB*-genes are present (data not shown), so that it is conceivable, although far to be proved, that RbsB may account for the internalization of AI-2 in a number of species lacking LsrB.

### Host specificity of *lsrB*-positive bacteria

Interestingly, bacteria that possess a Lsr-receptor complex are mostly associated with warm-blooded animals. In fact, this ABC-transporter could not be located in the *luxS*-positive bacteria *S. bongori*, which unlike other *Salmonella *spp. is usually associated with cold-blooded organisms [53], or in *B. subtilis*, which is a soil organism not considered to be an animal pathogen [54]. Exceptions are represented by the human pathogens *S. boydii *and *S. sonnei *in which the *lsr*-genes are absent [55] and insect pathogens *B. thuringensis *and *P. luminescens *which conversely feature the Lsr-complex. The case of *P. luminescens *is of particular significance since in this bacterium the presence of the *lsr*-locus seems restricted to those strains (i.e., TT01, Hb, and C1) forming a mutualistic consortium with entomopathogenic nematode *Heterorhabditis bacteriophora*, but shows ample gene deletions in strains associated with other nematode hosts or in related *Xenorhabdus *species suggesting that the *lsr *locus is strongly involved in the bacterial association with *H. bacteriophora *[56]. Altogether this data strongly suggest that AI-2 may be a relevant signal for many invasive bacteria and that the Lsr-receptor complex may generally play an important role in host colonization.

### Orphan receptors

**Figure 5:**
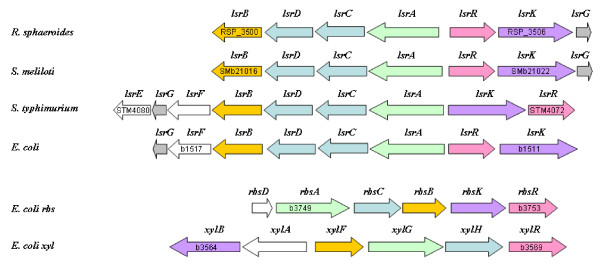
**Comparison of the operons coding for the Lsr-like receptors in the *luxS*-negative α-proteobacteria *R. sphaeroides *2.4.1 and *S. meliloti *1021 with the *lsr *cluster of AI-2 producers *S. typhimurium *and *E. coli *K12**. The *lsr*-like operons are located on the highly variable chromosome II of *R. sphaeroides *2.4.1 (accession number NC_007494) and on the pSymB megaplasmid of *S. meliloti *1021 (NC_003078) respectively. The transporter core is composed of a periplasmic AI-2 binding protein (LsrB), two hydrophobic proteins which for an homodimeric transmembrane channel (LsrC and LsrD) and a hydrophilic ATP-binding protein (LsrA). The expression of the *lsr *operon is controlled by repressor protein LsrR and AI-2 kinase LsrK which is responsible for the production of phospho-AI-2, the *lsr *operon inducer. In *S. typhimurium *(NC_003197), the LsrF and LsrG proteins are involved in modifying phospho-AI-2 [21]. For comparison ribose and xylose ABC transporters of *E. coli *K12 (NC_000913) are shown below. Proteins with the same function are coded with the same color. As point of reference, locus tags for selected genes are marked inside the arrows.

An unexpected result of our study was the discovery of a region with high homology to the *lsr *operon (*lsrBDCARKG*) (Fig. 5) on chromosome II of two of the three sequenced strains of the free-living photosynthetic bacterium *R. sphaeroides *and on plasmid pSymB of nitrogen-fixing plant symbiont *S. meliloti *1021. Both bacteria belong to the α-subdivision of Proteobacteria that regenerates homocysteine not by the means of the AMC, but directly from SAH using a one-step reaction catalyzed by SAH hydrolase. Since they are devoid of the LuxS enzyme they can not produce the AI-2 signal by themselves and are thus in principle dumb in the QS-2 language. In both bacteria the genes coding for the Lsr receptor complex are situated not on the main chromosome, but either on a megaplasmid mainly devoted at the codification of solute uptake systems in *S. meliloti *[57] or on a smaller chromosome with much lower information content density than the main chromosome, which is thought to provide highly adaptable organisms such as *R. sphaeroides *with genetic "safe zones" that allow DNA to change without affecting the organism itself [58]. The phylogeny of the *lsrB *gene (Fig 3) suggest that the AI-2 receptor complex in *R. sphaeroides and S. meliloti *was eventually acquired horizontally from unrelated enterobacterial species, such as e.g. *K. pneumoniae *which occurs as free-living diazotrophs in soil, plant endophytes, and as opportunistic human pathogens [59,60]. It is conceivable that *K. pneumoniae *acquired the Lsr receptor from other endotherm associated Enterobacteria and passed it to cohabitant nitrogen fixing bacteria in soil. Whether the Lsr-complex in *R. sphaeroides *and *S. meliloti *really enables them to react to the AI-2 signal still has to be proven experimentally, but is tempting to speculate that these α-Proteobacteria may use this receptor to eavesdrop on QS-2 communication employed by *K. pneumoniae *in soil.

### Dual pathways

Contrasting with other Actinobacteria that bear only the SAH hydrolase for the production of homocysteine using SAH as substrate, both *B. longum *and *B. adolescentis *also possess *luxS *in their genome. These species are key commensals of the human gastrointestinal tract and appear to have acquired *luxS *by horizontal gene transfer in the gut from other Gram-positive endotherm-associated bacteria (Fig 2). The presence of the other genes coding for the complete AMC and absence of *lsrB *from the genome of *Bifidobacterium *suggest that the additional occurrence of LuxS in these strains is not aimed to AI-2 production (albeit an homolog of SMU.408 of *S. mutans *is present), but represents only a redundant metabolic pathway for the recycling of homocysteine as proposed previously [61]. However, the possibility that an eventual AI-2 signal may be used for one-way interspecies communication can not be discounted.

Another non-pathogenic species, *E. blattae*, was also identified to have both pathways [19], but given its taxonomical classification among Enterobacteriaceae, here the horizontal gene transfer most probably involved *sahH *instead of *luxS*. As for *Bifidobacterium*, also in *E. blattae *the physiological significance of the double SAH detoxification pathway still awaits clarification.

### Phylogenetic analysis

Previous studies have assessed that at the level of bacterial phyla the phylogenetic tree based on *luxS *largely corresponds to the one constructed using 16S ribosomal RNA, demonstrating that the former gene is ancestral in groups like Proteobacteria and Firmicutes. ε-proteobacterium *H. pylori*, spirochete *B. burgdorferi *and actinobacterium *B. longum*, which clustered with Firmicutes in the *luxS *tree, represented the exception to this criterion indicating that horizontal gene transfer has occurred in these species [62]. Our result confirm these findings and suggest that also *B. adolescentis*, or more probably a common *Bifidobacterium *ancestor, may have undergone an HGT (Fig. 2). In order to understand if and how the AI-2 receptors coevolved with the AI-2 synthesis gene, we first analyzed the distribution of the recognized receptor genes on the entire *luxS *phylogenetic tree (Fig. 2), then compared the tree based on the *lsrB *receptor with the *luxS *and *rpoB *trees of the matching strains (Fig. 4). As previously reported the presence of the LuxP receptor is fundamentally limited to Vibrionaceae [19] and its evolution corroborates the fact that this kind of receptor has been constantly present during the evolution of this family of marine bacteria (Fig. 2). On the other hand the Lsr complex is widespread (but not ubiquitous) in Enterobacteriaceae and Bacillaceae, as well as present in two species belonging to the family Pasteurellaceae (i.e., *P. multocida *and *H. somnus*). Despite the fact that these bacterial groups are fairly separate on an evolutionary level, the branching pattern of the *lsrB *tree is mostly concordant with the corresponding tree based on *luxS *and housekeeping gene *rpoB *(Fig. 4), implicating that this kind of AI-2 receptor genes are probably orthologs that have co-evolved with the AI-2 producer gene. An alternative hypothesis postulating the Lsr receptor complex being a paralog of closely related ABC transporter, such as ribose transporter Rbs, is not supported by the available data which demonstrate that *rbs *genes of distant related species are more similar to each other, than equivalent *rbs *and *lsr *genes in the same species (data not shown). As expected from previous studies on ABC systems [63], also shuffling of genes between different transporters is not supported by phylogenetic analysis (data not shown).

The fact that in no bacteria the components of the AI-2 receptor and signal transduction pathway are found clustered together with *luxS *in the genome further suggests that the QS-2 system was built upon a pre-existent general metabolic function by some bacteria only, which may help to explain why it is not an universal feature of all species.

## Conclusion

Although in order to be able to detect the AI-2 signal and to react in a QS-2 dependent manner bacteria must carry an appropriate receptor for the autoinducer, in the light of our analysis it is apparent that there is still a common misconception in the literature leading to the conclusion that the mere presence of the *luxS *gene is a sufficient condition to deduce the existence of type-2 quorum sensing. In these works the functionality of LuxS is usually demonstrated by the ability of the wildtype strain supernatants to stimulate luminescence in AI-2 reporter strain *V. harveyi *BB170, by the competence of the species-specific *luxS *gene to induce AI-2 production when introduced in a *luxS*-negative strain such as *E. coli *DH5α or by *in trans *complementation of *luxS*-mutants to restore the original phenotype. It is critical to understand that these tests are in any case insufficient to demonstrate the existence of a QS-2 based communication. Even for bacteria which are devoid of a functional QS-2, the *V. harveyi *reporter assay will result in a simple test of the activity of the AMC, as AI-2 is produced spontaneously from DPD [33,34], which is a by-product as of this metabolic cycle (Fig. 1).

The complementation of AI-2 production in *E. coli *DH5α, which has a defective *luxS *due to a frameshift-mutation [64], using a foreign *luxS*-gene merely proves its functionality, but does not give any indication about the production and most importantly the detection of AI-2 in the original organism. Finally, any defect induced by the *luxS *mutation in the methionine recycling pathway (Fig. 1) will be in most cases fully restored by the straightforward genetic complementation [65].

It is thus imperative for QS-2 studies to provide evidence for AI-2 receptor also include a chemical complementation of the *luxS *mutant [31], either by using AI-2 conditioned supernatants or by using the chemically defined AI-2 molecule [32]. New criteria for establishing the functionality of QS-2 in a given species should include also identification and mutational analysis of putative AI-2 receptors, RNA/protein expression patterns in presence/absence of the AI-2 signal and, if the latter is transported inside the cell as in the case of the Lsr receptor complex, AI-2 consumption profiles. In bacteria lacking the Lsr-operon this would enable assessment of the role as alternative AI-2 receptors for related ABC-transporters, e.g., ribose Rbs receptor complex [66] or xylose Xyl receptor complex [67,68]. An alternative approach may consist in the genetic complementation of the sole metabolic function of the defective *luxS *gene by replacing it with an exogenous *sahH *gene encoding the SAH hydrolase, which is part of the other known S-adenosylhomocysteine detoxification pathway. In this way it is possible to repair the function of the AMC in *E. coli *without restoring a potential AI-2 production [69].

In any case the possibility that some bacteria lacking an AI-2 receptor either produce AI-2 for interference with signaling of other bacteria or that inadvertently produced AI-2 is detected by other bacteria can not be discarded and may have important ecological implications. Yet, a disease control strategy aimed to interfere with intra- or interspecific QS-2 based crosstalk between bacteria (the so-called quorum quenching), or as a means to enhance QS-2 in beneficial bacteria, would have hope of success only for those species which carry a functional AI-2 receptor. As suggested by Xavier and Bassler [8], this may be true for many human pathogenic bacteria (e.g., *E. coli*, *K. pneumoniae*, *Shigella *spp., *Salmonella *spp., *Yersinia *spp. and *Bacillus cereus *spp.) for which the presence of the Lsr-receptor complex was confirmed herein. On the other hand, the prospective of infection control using AI-2 based quorum quenching is less concrete for plant diseases, since no AI-2 receptor was unambiguously confirmed in specialized plant pathogens, meaning that in these bacteria the role of *luxS *is most probably limited to metabolism.

## Authors' contributions

FR initiated the study, conducted the data mining work and contributed to writing the manuscript. BD supervised the study and contributed to writing the manuscript. Both authors read and approved the final manuscript.

## Supplementary Material

Additional file 1 **Table 1s.** Relationship between the presence of the *luxS *gene,  genes encoding known AI-2 receptors and QS-2 dependent behavior in studies performed on bacteria,  with short description of the results obtained in each work cited. Click here for file

Additional file 2 **Figure 1s.** Phylogenetic relationships among sequenced *Bacillus *species on the basis of complete *luxS *sequences and the presence of the *lsrB *receptor in the respective genomes. Click here for file

## References

[B1] Whitehead NA, Barnard AM, Slater H, Simpson NJ, Salmond GP (2001). Quorum-sensing in Gram-negative bacteria. FEMS Microbiol Rev.

[B2] Cao JG, Meighen EA (1989). Purification and structural identification of an autoinducer for the luminescence system of *Vibrio harveyi*. J Biol Chem.

[B3] Engebrecht J, Nealson K, Silverman M (1983). Bacterial bioluminescence: isolation and genetic analysis of functions from *Vibrio fischeri*. Cell.

[B4] McKenney D, Brown KE, Allison DG (1995). Influence of *Pseudomonas aeruginosa *exoproducts on virulence factor production in *Burkholderia cepacia*: evidence of interspecies communication. J Bacteriol.

[B5] Steidle A, Sigl K, Schuhegger R, Ihring A, Schmid M, Gantner S, Stoffels M, Riedel K, Givskov M, Hartmann A, Langebartels C, Eberl L (2001). Visualization of N-acylhomoserine lactone-mediated cell-cell communication between bacteria colonizing the tomato rhizosphere. Appl Environ Microbiol.

[B6] Bassler BL (2002). Small talk. Cell-to-cell communication in bacteria. Cell.

[B7] Bassler BL, Wright M, Showalter RE, Silverman MR (1993). Intercellular signalling in *Vibrio harveyi*: sequence and function of genes regulating expression of luminescence. Mol Microbiol.

[B8] Xavier KB, Bassler BL (2005). Interference with AI-2-mediated bacterial cell-cell communication. Nature.

[B9] Schauder S, Shokat K, Surette MG, Bassler BL (2001). The LuxS family of bacterial autoinducers: biosynthesis of a novel quorum sensing signal molecule. Mol Microbiol.

[B10] Winans SC (2002). Bacterial Esperanto. Nature Struct Biol.

[B11] Winzer K, Hardie KR, Williams P (2003). LuxS and autoinducer-2: their contribution to quorum sensing and metabolism in bacteria. Adv Appl Microbiol.

[B12] Freeman JA, Bassler BL (1999). Sequence and function of LuxU: a two-component phosphorelay protein that regulates quorum sensing in *Vibrio harveyi*. J Bacteriol.

[B13] Freeman JA, Bassler BL (1999). A genetic analysis of the function of LuxO, a two-component response regulator involved in quorum sensing in *Vibrio harveyi*. Mol Microbiol.

[B14] Reading NC, Sperandio V (2006). Quorum sensing: the many languages of bacteria. FEMS Microbiol Lett.

[B15] Miyamoto CM, Lin YH, Meighen EA (2000). Control of bioluminescence in *Vibrio fischeri *by the LuxO signal response regulator. Mol Microbiol.

[B16] Makino K, Oshima K, Kurokawa K, Yokoyama K, Uda T, Tagomori K, Iijima Y, Najima M, Nakano M, Yamashita A, Kubota Y, Kimura S, Yasunaga T, Honda T, Shinagawa H, Hattori M, Iida T (2003). Genome sequence of *Vibrio parahaemolyticus*: a pathogenic mechanism distinct from that of *V. cholerae*. Lancet.

[B17] Chen CY, Wu KM, Chang YC, Chang CH, Tsai HC, Liao TL, Liu YM, Chen HJ, Shen AB, Li JC, Su TL, Shao CP, Lee CT, Hor LI, Tsai SF (2003). Comparative genome analysis of *Vibrio vulnificus*, a marine pathogen. Genome Res.

[B18] Kasai S (2006). Freshwater bioluminescence in *Vibrio albensis *(*Vibrio cholerae *biovar *albensis*) NCIMB 41 is caused by a two-nucleotide deletion in *luxO*. J Biochem.

[B19] Sun J, Daniel R, Wagner-Döbler I, Zeng AP (2004). Is autoinducer-2 a universal signal for interspecies communication: a comparative genomic and phylogenetic analysis of the synthesis and signal transduction pathways. BMC Evol Biol.

[B20] Taga ME, Semmelhack JL, Bassler BL (2001). The LuxS dependent autoinducer AI-2 controls the expression of an ABC-transporter that functions in AI-2 uptake in *Salmonella typhimurium*. Mol Microbiol.

[B21] Xavier KB, Bassler BL (2005). Regulation of uptake and processing of the quorum-sensing autoinducer AI-2 in *Escherichia coli*. J Bacteriol.

[B22] Taga ME, Miller ST, Bassler BL (2003). Lsr-mediated transport and processing of AI-2 in *Salmonella typhimurium*. Mol Microbiol.

[B23] Winzer K, Hardie KR, Williams P (2002). Bacterial cell-to-cell communication: sorry, can't talk now – gone to lunch!. Curr Opin Microbiol.

[B24] Winzer K, Hardie KR, Burgess N, Doherty N, Kirke DF, Holden MTG, Linforth R, Cornell KA, Taylor AJ, Hill PJ, Williams P (2002). LuxS: its role in central metabolism and the in vitro synthesis of 4-hydroxy-5-methyl-3(2H)-furanone. Microbiology.

[B25] Shao H, James D, Lamont RJ, Demuth DR (2007). Differential interaction of *Aggregatibacter *(*Actinobacillus*) *actionmycetemcomitans *LsrB and RbsB proteins with Autoinducer 2. J Bacteriol.

[B26] Surette MG, Bassler BL (1998). Quorum sensing in *Escherichia coli *and *Salmonella typhimurium*. Proc Natl Acad Sci USA.

[B27] Surette MG, Bassler BL (1999). Regulation of autoinducer production in *Salmonella typhimurium*. Mol Microbiol.

[B28] Ren D, Sims JJ, Wood TK (2001). Inhibition of biofilm formation and swarming of *Escherichia coli *by (5Z)-4-bromo-5-(bromomethylene)-3-butyl-2(5H)-furanone. Environ Microbiol.

[B29] González Barrios AF, Zuo R, Hashimoto Y, Yang L, Bentley WE, Wood TK (2006). Autoinducer 2 controls biofilm formation in *Escherichia coli *through a novel motility quorum-sensing regulator (MqsR, B3022). J Bacteriol.

[B30] DeLisa MP, Wu CF, Wang L, Valdes JJ, Bentley WE (2001). DNA microarray-based identification of genes controlled by autoinducer 2-stimulated quorum sensing in *Escherichia coli*. J Bacteriol.

[B31] Vendeville A, Winzer K, Heurlier K, Tang CM, Hardie KR (2005). Making 'sense' of metabolism: autoinducer-2, LuxS and pathogenic bacteria. Nature Rev Microbiol.

[B32] Meijler MM, Kaufmann GF, Hom LG, McKenzie K, Sun C, Moss JA, Matsushita M, Janda KD (2004). Synthesis and biological validation of a ubiquitous quorum-sensing molecule. Angew Chem Int Ed Engl.

[B33] Chen X, Schauder S, Potier N, Van Dorssealaer A, Pelczer I, Bassler BL, Hughson FM (2002). Structural identification of bacterial quorum-sensing signal containing boron. Nature.

[B34] Miller ST, Xavier KB, Campagna S, Taga ME, Semmelhack MF, Bassler BL, Hughson FM (2004). *Salmonella typhimurium *recognizes a chemically distinct form of the bacterial quorum-sensing signal AI-2. Mol Cell.

[B35] Cannon SB, Young ND (2003). OrthoParaMap: distinguishing orthologs from paralogs by integrating comparative genome data and gene phylogenies. BMC Bioinformatics.

[B36] Thompson JD, Higgins DG, Gibson TJ (1994). CLUSTAL W: improving the sensitivity of progressive multiple sequence alignment through sequence weighting, position-specific gap penalties and weight matrix choice. Nucleic Acids Res.

[B37] Tamura K, Dudley J, Nei M, Kumar S (2007). MEGA4: Molecular Evolutionary Genetics Analysis (MEGA) software version 4.0. Mol Biol Evol.

[B38] Kim SY, Lee SE, Kim YR, Kim CM, Ryu PY, Choy HE, Chung SS, Rhee JH (2003). Regulation of *Vibrio vulnificus *virulence by the LuxS quorum-sensing system. Mol Microbiol.

[B39] Bassler BL, Greenberg EP, Stevens AM (1997). Cross-species induction of luminescence in the quorum-sensing bacterium *Vibrio harveyi*. J Bacteriol.

[B40] Lilley BN, Bassler BL (2000). Regulation of quorum sensing in *Vibrio harveyi *by LuxO and Sigma-54. Mol Microbiol.

[B41] Blevins JS, Revel AT, Caimano MJ, Yang XF, Richardson JA, Hagman KE, Norgard MV (2004). The *luxS *gene is not required for *Borrelia burgdorferi *tick colonization, transmission to a mammalian host, or induction of disease. Infect Immun.

[B42] Lapidus A, Goltsman E, Auger S, Galleron N, Ségurens B, Dossat C, Land ML, Broussolle V, Brillard J, Guinebretière MH, Sanchis V, Nguen-The C, Lereclus D, Richardson P, Wincker P, Weissenbach J, Ehrlich SD, Sorokin A (2008). Extending the *Bacillus cereus *group genomics to putative food-borne pathogens of different toxicity. Chem Biol Interact.

[B43] Wiezer A (2004). Entschlüsselung der Genomsequenz von *Escherichia blattae *und komparative Bioinformatik mikrobieller Genome. Ph D thesis.

[B44] Federle MJ, Bassler BL (2003). Interspecies communication in bacteria. J Clin Invest.

[B45] Fong KP, Chung WO, Lamont RJ, Demuth DR (2001). Intra- and interspecies regulation of gene expression by *Actinobacillus actinomycetemcomitans *LuxS. Infect Immun.

[B46] McNab R, Ford SK, El-Sabaeny A, Barbieri B, Cook GS, Lamont RJ (2003). LuxS-based signaling in *Streptococcus gordonii*: autoinducer-2 controls carbohydrate metabolism and biofilm formation with *Porphyromonas gingivalis*. J Bacteriol.

[B47] Eilers H, Pernthaler J, Glockner FO, Amann R (2000). Culturability and in situ abundance of pelagic bacteria from the North Sea. Appl Environ Microbiol.

[B48] Weidner S, Arnold W, Stackebrandt E, Pühler A (2000). Phylogenetic analysis of bacterial communities associated with leaves of the seagrass *Halophila stipulacea *by a culture-independent small-subunit rRNA gene approach. Microb Ecol.

[B49] Yoshida A, Ansai T, Takehara T, Kuramitsu HK (2005). LuxS-based signaling affects *Streptococcus mutans *biofilm formation. Appl Environ Microbiol.

[B50] Sztajer H, Lemme A, Vilchez R, Schulz S, Geffers R, Ying Yin Yip C, Levesque CM, Cvitkovitch DG, Wagner-Döbler I (2008). Autoinducer-2-regulated genes in *Streptococcus mutans *UA159 and global metabolic effect of the *luxS *mutation. J Bacteriol.

[B51] Stevenson B, von Lackum K, Wattier RL, McAlister JD, Miller JC, Babb K (2003). Quorum sensing by the Lyme disease spirochete. Micr Inf.

[B52] Shao H, Lamont RJ, Demuth DR (2007). Autoinducer 2 is required for biofilm growth of *Aggregatibacter *(*Actinobacillus*) *actionmycetemcomitans*. Infect Immun.

[B53] Chan K, Bake S, Kim CC, Detweiler CS, Dougan G, Falkow S (2003). Genomic comparison of *Salmonella enterica *serovars and *Salmonella bongori *by use of *an S. enterica *serovar *typhimurium *DNA Microarray. J Bacteriol.

[B54] Madigan M, Martinko J (2005). Brock Biology of Microorganisms.

[B55] Litwin CM, Storm AL, Chipowsky S, Ryan KJ (1991). Molecular epidemiology of Shigella infections: plasmid profiles, serotype correlation, and restriction endonuclease analysis. J Clin Microbiol.

[B56] Gaudriault S, Duchaud E, Lanois A, Canoy AS, Bourot S, DeRose R, Kunst F, Boemare N, Givaudan A (2006). Whole-genome comparison between *Photorhabdus *strains to identify genomic regions involved in the specificity of nematode interaction. J Bacteriol.

[B57] Finan TM, Weidner S, Wong K, Buhrmester J, Chain P, Vorholter FJ, Hernandez-Lucas I, Becker A, Cowie A, Gouzy J, Golding B, Puhler A (2001). The complete sequence of the 1,683-kb pSymB megaplasmid from the N2-fixing endosymbiont *Sinorhizobium meliloti*. Proc Natl Acad Sci USA.

[B58] Choudhary M, Zanhua X, Fu YX, Kaplan S (2007). Genome analyses of three strains of *Rhodobacter sphaeroides*: evidence of rapid evolution of chromosome II. J Bacteriol.

[B59] Dong Y, Chelius MK, Brisse S, Kozyrovska N, Kovtunovych G, Podschun R, Triplett EW (2003). Comparisons between two *Klebsiella*: the plant endophyte *K. pneumoniae *342 and a clinical isolate, *K. pneumoniae *MGH78578. Symbiosis.

[B60] Postgate J (1998). Nitrogen fixation.

[B61] Schell MA, Karmirantzou M, Snel B, Vilanova D, Berger B, Pessi G, Zwahlen MC, Desiere F, Bork P, Delley M, Pridmore RD, Arigoni F (2002). The genome sequence of *Bifidobacterium longum *reflects its adaptation to the human gastrointestinal tract. Proc Natl Acad Sci USA.

[B62] Lerat E, Moran NA (2004). The evolutionary history of quorum-sensing systems in bacteria. Mol Biol Evol.

[B63] Dassa E, Hofnung M, Paulsen IT, Saier MH (1999). The *Escherichia coli *ABC transporters: an update. Mol Microbiol.

[B64] Surette MG, Miller MB, Bassler BL (1999). Quorum sensing in *Escherichia coli*, *Salmonella typhimurium*, and *Vibrio harveyi*: a new family of genes responsible for autoinducer production. Proc Natl Acad Sci USA.

[B65] McDougald D, Rice SA, Kjelleberg S (2007). Bacterial quorum sensing and interference by naturally occurring biomimics. Anal Bioanal Chem.

[B66] Park Y, Park C (1999). Topology of RbsC, a membrane component of the ribose transporter, belonging to the AraH superfamily. J Bacteriol.

[B67] Rosenfeld SA, Tevis PE, Ho NWY (1984). Cloning and characterization of the *xyl *genes from *Escherichia coli*. Mol Gen Genet.

[B68] James D, Shao H, Lamont RJ, Demuth DR (2006). The *Actinobacillus actinomycetemcomitans *ribose binding protein RbsB interacts with cognate and heterologous autoinducer 2 signals. Infect Immun.

[B69] Walters M, Sircili MP, Sperandio V (2006). AI-3 synthesis is not dependent on *luxS *in *Escherichia coli*. J Bacteriol.

[B70] Fong KP, Gao L, Demuth DR (2003). *luxS *and *arcB *control aerobic growth of *Actinobacillus actinomycetemcomitans *under iron limitation. Infect Immun.

[B71] Jones MB, Blaser MJ (2003). Detection of a *luxS *-signaling molecule in *Bacillus anthracis*. Infect Immun.

[B72] Auger S, Krin E, Aymerich S, Gohar M (2006). Autoinducer 2 affects biofilm formation by *Bacillus cereus*. Appl Environ Microbiol.

[B73] Sperandio V, Torres AG, Giron JA, Kaper JB (2001). Quorum sensing is a global regulatory mechanism in enterohemorrhagic *Escherichia coli *O157:H7. J Bacteriol.

[B74] Wang L, Li J, March JC, Valdes JJ, Bentley WE (2005). *luxS *-dependent gene regulation in *Escherichia coli *K-12 revealed by genomic expression profiling. J Bacteriol.

[B75] Inzana TJ, Glindemann GE, Larson J, Siddaramappa S (2003). Biofilm formation by the bovine specific pathogen *Haemophilus somnus*. Biofilms 2003, ASM Conferences: 1–7 November 2003; Victoria, British Columbia.

[B76] Balestrino D, Haagensen JA, Rich C, Forestier C (2005). Characterization of type 2 quorum sensing in *Klebsiella pneumoniae *and relationship with biofilm formation. J Bacteriol.

[B77] Malotta RJ, Loa RYC (2002). Studies on the production of quorum-sensing signal molecules in *Mannheimia haemolytica *A1 and other Pasteurellaceae species. FEMS Microbiol Lett.

[B78] Krin E, Chakroun N, Turlin E, Givaudan A, Gaboriau F, Bonne I, Rousselle JC, Frangeul L, Lacroix C, Hullo MF, Marisa L, Danchin A, Derzelle S (2006). Pleiotropic role of quorum-sensing autoinducer 2 in *Photorhabdus luminescens*. Appl Environ Microbiol.

[B79] Brandl MT, Miller WG, Bates AH, Mandrell RE (2005). Production of autoinducer 2 in *Salmonella enterica *serovar *Thompson *contributes to its fitness in chickens but not on cilantro leaf surfaces. Appl Environ Microbiol.

[B80] Day WA, Maurelli AT (2001). *Shigella flexneri *LuxS quorum-sensing system modulates *virB *expression but is not essential for virulence. Infect Immun.

[B81] Croxatto A, Pride J, Hardman A, Williams P, Cámara M, Milton DL (2004). A distinctive dual-channel quorum sensing system operates in *Vibrio anguillarum*. Mol Microbiol.

[B82] Miller MB, Skorupski K, Lenz DH, Taylor RK, Bassler BL (2002). Parallel quorum sensing systems converge to regulate virulence in *Vibrio cholerae*. Cell.

[B83] Bassler BL, Wright M, Silverman MR (1994). Multiple signalling systems controlling expression of luminescence in *Vibrio harveyi*: sequence and function of genes encoding a second sensory pathway. Mol Microbiol.

[B84] Lupp C, Ruby EG (2004). *Vibrio fischeri *LuxS and AinS: comparative study of two signal synthases. J Bacteriol.

[B85] Henke JM, Bassler BL (2004). Quorum Sensing Regulates Type III Secretion in *Vibrio harveyi *and *Vibrio parahaemolyticus*. J Bacteriol.

[B86] Jarrett CO, Deak E, Isherwood KE, Oyston PC, Fischer ER, Whitney AR, Kobayashi SD, DeLeo FR, Hinnebusch BJ (2004). Transmission of *Yersinia pestis *from an infectious biofilm in the flea vector. J Infect Dis.

[B87] Bobrov AG, Abu Khweek A, Perry RD, Parrish KD, Fetherston JD, Bearden SW, Perry RD, Fetherston JD (2007). Functional quorum sensing systems affect biofilm formation and protein expression in *Yersinia pestis*. The genus Yersinia: from genomics to function.

[B88] Sheehan BJ, Bosse JT, Beddek AJ, Rycroft AN, Kroll JS, Langford PR (2003). Identification of *Actinobacillus pleuropneumoniae *genes important for survival during infection in its natural host. Infect Immun.

[B89] Stevenson B, Babb K (2002). LuxS-mediated quorum sensing in *Borrelia burgdorferi*, the Lyme disease spirochete. Infect Immun.

[B90] Stevenson B, von Lackum K, Wattier RL, McAlister JD, Miller JC, Babb K (2003). Quorum sensing by the Lyme disease spirochete. Micr Infect.

[B91] Babb K, von Lackum K, Wattier RL, Riley SP, Stevenson B (2005). Synthesis of autoinducer-2 by the Lyme disease spirochete, *Borrelia burgdorferi*. J Bacteriol.

[B92] Hübner A, Revel AT, Nolen DM, Hagman KE, Norgard MV (2003). Expression of a *luxS *gene is not required for *Borrelia burgdorferi *infection of mice via needle inoculation. Infect Immun.

[B93] Carter GP, Purdy D, Williams P, Minton NP (2005). Quorum sensing in *Clostridium difficile*: analysis of a *luxS*-type signalling system. J Med Microbiol.

[B94] Lee AS, Song KP (2005). LuxS/autoinducer-2 quorum sensing molecule regulates transcriptional virulence gene expression in *Clostridium difficile*. Biochem Biophys Res Commun.

[B95] Rezzonico F, Duffy B (2007). The role of *luxS *in the fire blight pathogen *Erwinia amylovora *is limited to metabolism and does not involve quorum sensing. Mol Plant-Microbe Interact.

[B96] Mohammadi M, Geider K (2007). Autoinducer-2 of the fire blight pathogen *Erwinia amylovora *and other plant-associated bacteria. FEMS Microbiol Lett.

[B97] Forsyth MH, Cover TL (2000). Intercellular communication in *Helicobacter pylori*: *luxS *is essential for the production of an extracellular signaling molecule. Infect Immun.

[B98] Loh JT, Forsyth MH, Cover TL (2004). Growth phase regulation of *flaA *expression in *Helicobacter pylori *is *luxS *dependent. Infect Immun.

[B99] Joyce EA, Bassler BL, Wright A (2000). Evidence for a signaling system in *Helicobacter pylori*: detection of a *luxS*-encoded autoinducer. J Bacteriol.

[B100] Osaki T, Hanawa T, Manzoku T, Fukuda M, Kawakami H, Suzuki H, Yamaguchi H, Yan X, Taguchi H, Kurata S, Kamiya S (2006). Mutation of *luxS *affects motility and infectivity of *Helicobacter pylori *in gastric mucosa of a Mongolian gerbil model. J Med Microbiol.

[B101] Lee W-K, Ogura K, Loh JT, Cover TL, Berg DE (2006). Quantitative effect of *luxS *gene inactivation on the fitness of *Helicobacter pylori*. Appl Environ Microbiol.

[B102] Lebeer S, De Keersmaecker SC, Verhoeven TL, Fadda AA, Marchal K, Vanderleyden J (2007). Functional analysis of *luxS *in the probiotic strain *Lactobacillus rhamnosus *GG reveals a central metabolic role important for growth and biofilm formation. J Bacteriol.

[B103] Sela S, Frank S, Belausov E, Pinto R (2006). A mutation in the *luxS *gene influences *Listeria monocytogenes *biofilm formation. Appl Environ Microbiol.

[B104] Challan-Belval S, Gal L, Margiewes S, Garmyn D, Piveteau P, Guzzo J (2006). Assessment of the roles of LuxS, S-ribosyl homocysteine, and an autoinducer-2 in cell attachment during biofilm formation by *Listeria monocytogenes *EGD-e. Appl Environ Microbiol.

[B105] Winzer K, Sun Y-H, Green A, Delory M, Blackley D, Hardie KR, Baldwin TJ, Tang C (2002). Role of *Neisseria meningitidis luxS *in cell-to-cell signalling and bacteremic infection. Infect Immun.

[B106] Schauder S, Penna L, Ritton A, Manin C, Parker F, Renauld-Mongenie G (2005). Proteomics analysis by two-dimensional differential gel electrophoresis reveals the lack of a broad response of *Neisseria meningitidis *to in vitro-produced AI-2. J Bacteriol.

[B107] Schneider R, Lockatell CV, Johnson D, Belas R (2002). Detection and mutation of a *luxS *-encoded autoinducer in *Proteus mirabilis*. Microbiology.

[B108] van Houdt R, Moons P, Jansen A, Vanoirbeek K, Michiels CW (2006). Isolation and functional analysis of *luxS *in *Serratia plymuthica *RVH1. FEMS Microbiol Lett.

[B109] Doherty N, Holden MT, Qazi SN, Williams P, Winzer K (2006). Functional analysis of *luxS *in *Staphylococcus aureus *reveals a role in metabolism but not quorum sensing. J Bacteriol.

[B110] Merritt J, Qi F, Goodman SD, Anderson MH, Shi W (2003). Mutation of *luxS *affects biofilm formation in *Streptococcus mutans*. Infect Immun.

[B111] Wen ZT, Burne RA (2002). Functional genomics approach to identifying genes required for biofilm development by *Streptococcus mutans*. Appl Environ Microbiol.

[B112] Siller M, Janapatla RP, Charpentier E (2006). Regulation of virulence factor expression by AI-2/*luxS *signaling in *Streptococcus pyogenes *is serotype-dependent. 2nd FEMS Congress of European Microbiologist: 4–8 July 2006; Madrid, Spain.

[B113] Marouni MJ, Sela S (2003). The *luxS *gene of *Streptococcus pyogenes *regulates expression of genes that affect internalization by epithelial cells. Infect Immun.

[B114] Lyon WR, Madden JC, Levin JC, Stein JL, Caparon MG (2001). Mutation of *luxS *affects growth and virulence factor expression in *Streptococcus pyogenes*. Mol Microbiol.

[B115] Rickard AH, Palmer RJ, Blehert DS, Campagna SR, Semmelhack MF, Egland PG, Bassler BL, Kolenbrander PE (2006). Autoinducer 2: a concentration-dependent signal for mutualistic bacterial biofilm growth. Mol Microbiol.

[B116] Lombardia E, Rovetto AJ, Arabolaza AL, Grau RR (2006). A LuxS-dependent cell-to-cell language regulates social behavior and development in *Bacillus subtilis*. J Bacteriol.

[B117] Elvers KT, Park SF (2002). Quorum sensing in *Campylobacter jejuni*: detection of a *luxS *encoded signaling molecule. Microbiology.

[B118] Jeon B, Itoh K, Misawa N, Ryu S (2003). Effects of quorum sensing on *flaA *transcription and autoagglutination in *Campylobacter jejuni*. Microbiol Immunol.

[B119] Ohtani K, Hayashi H, Shimizu T (2002). The *luxS *gene is involved in cell-cell signalling for toxin production in *Clostridium perfringens*. Mol Microbiol.

[B120] Kaper JB, Sperandio V (2005). Bacterial cell-to-cell signaling in the gastrointestinal tract. Infect Immun.

[B121] Coulthurst SJ, Lilley KS, Salmond GP (2006). Genetic and proteomic analysis of the role of *luxS *in the enteric phytopathogen, *Erwinia carotovora*. Mol Plant Pathol.

[B122] Laasik E, Andresen L, Mäe A (2006). Type II quorum sensing regulates virulence in *Erwinia carotovora *ssp. *carotovora*. FEMS Microbiol Lett.

[B123] Vezzi A, Campanaro S, D'Angelo M, Simonato F, Vitulo N, Lauro FM, Cestaro A, Malacrida G, Simionati B, Cannata N, Romualdi C, Bartlett DH, Valle G (2005). Life at depth: *Photobacterium profundum *genome sequence and expression analysis. Science.

[B124] Chung W, Park Y, Lamont RJ, McNab R, Barbieri B, Demuth DR (2001). A signaling system in *Porphyromonas gingivalis *based on a LuxS protein. J Bacteriol.

[B125] Burgess NA, Kirke DF, Williams P, Winzer K, Hardie KR, Meyers NL, Aduse-Opoku J, Curtis MA, Camara M (2002). LuxS-dependent quorum sensing in *Porphyromonas gingivalis *modulates protease and haemagglutinin activities but is not essential for virulence. Microbiology.

[B126] Yuan L, Hillman JD, Progulske-Fox A (2005). Microarray analysis of quorum-sensing-regulated genes in *Porphyromonas gingivalis*. Infect Immun.

[B127] James CE, Hasegawa Y, Park Y, Yeung V, Tribble GD, Kuboniwa M, Demuth DR, Lamont RJ (2006). LuxS involvement in the regulation of genes coding for hemin and iron acquisition systems in *Porphyromonas gingivalis*. Infect Immun.

[B128] Duan K, Dammel C, Stein J, Rabin H, Surette MG (2003). Modulation of *Pseudomonas aeruginosa *gene expression by host microflora through interspecies communication. Mol Microbiol.

[B129] Coulthurst SJ, Kurz CL, Salmond GPC (2004). *luxS *mutants of *Serratia *defective in autoinducer-2-dependent 'quorum sensing' show strain-dependent impacts on virulence and production of carbapenem and prodigiosin. Microbiology.

[B130] Bodor A, Elxnat B, Thiel V, Schulz S, Wagner-Döbler I (2008). Potential for *luxS *related signalling in marine bacteria and production of autoinducer-2 in the genus *Shewanella*. BMC Microbiology.

[B131] Xu L, Li H, Vuong C, Vadyvaloo V, Wang J, Yao Y, Otto M, Gao Q (2006). Role of the *luxS *quorum-sensing system in biofilm formation and virulence of *Staphylococcus epidermidis*. Infect Immun.

[B132] Ahmed NA, Petersen FC, Scheie AA (2007). AI-2 quorum sensing affects antibiotic susceptibility in *Streptococcus anginosus*. J Antimicrob Chemother.

[B133] Lönn-Stensrud J, Petersen FC, Benneche T, Scheie AA (2007). Synthetic bromated furanone inhibits autoinducer-2-mediated communication and biofilm formation in oral streptococci. Oral Microbiol Immunol.

[B134] Stroeher UH, Paton AW, Ogunniyi AD, Paton JC (2003). Mutation of *luxS *of *Streptococcus pneumoniae *affects virulence in a mouse model. Infect Immun.

