# Supplementary material for: Lack of genomic evidence of AI-2 receptors suggests a non-quorum sensing role for luxS in most bacteria
Source: BMC Microbiol. 2008 Sep 20;8:154. doi: 10.1186/1471-2180-8-154 (PMC2561040; doi:10.1186/1471-2180-8-154)
Supplement: Additional file 1 — Table 1s. Relationship between the presence of the luxS gene, genes encoding known AI-2 receptors and QS-2 dependent behavior in studies performed on bacteria, with short description of the results obtained in each work cited. [file 1471-2180-8-154-S1.pdf]

TABLE 1s. Relationship between the presence of the *luxS* gene, genes encoding known AI-2 receptors and QS-2 dependent behavior in studies performed on bacteria.

Strain	Talk[a] *luxS*	Listen[a] *luxP*	*lsrB*	Answer[b] AI-2 related phenotype	Chemical compl.[c]	Effect of AI-2/*luxS*	References
**a. Bacterial species carrying either a *luxP*- or *lsrB*-gene homolog in their genome**							
Actinobacillus actinomycetemcomitans	+	-	+	yes	+/SN	Induction of leukotoxic activity and increase in leukotoxin polypeptide by conditioned media of *luxS*[+] strains.	[45]
				yes	nd	Reduced growth in the mutant under aerobic, iron-limited conditions. AI-2 and ribose bind at the same site of RbsB.	[70]
				yes	+/AI-2	LsrB is present and may be involved in borate scavenging.	[68]
				yes<br>yes	+/AI-2	AI-2 is required for biofilm growth, *luxS* mutation is not complemented by SAH-hydrolase.	[25, 52]
Bacillus anthracis	+	-	+	yes	nd	Growth speed in heart infusion broth decreased in mutant, only *luxS* functionality and AI-2 production shown.	[71]
Bacillus cereus	+	-	+	yes	+/AI-2	Exogenous AI-2 has inhibitory effect on biofilm formation.	[72]
Bacillus thuringensis	+	-	+	nd	nd	No studies about QS-2 to date.	-
Desulfovibrio desulfuricans	-	(+)	-	nd	nd	Carries a LuxP homolog which is not flanked by a LuxQ homolog.	This work
Escherichia coli	+	-	+	yes	+/SN	Differential regulation of more > 400 genes demonstrated in *luxS* mutant.	[73]
				yes	+/SN	Differential regulation of 242 genes after supernatants addition.	[30]
				yes	+/SN	Lsr-dependent AI-2 uptake demonstrated.	[21]
				Q	nd	Role of *luxS* is dependent from glucose concentration. Most of the highly induced genes are related to AI-2 production and transport.	[74]
Haemophilus somnus	+	-	+	H	nd	Role in biofilm formation.	[75]
Klebsiella pneumoniae	+	-	+	H	nd	Positive role in formation of microcolonies, but not of mature biofilm. Only genetic, but no chemical complementation.	[76]
Pasteurella multocida	+	-	+	H	nd	Only presence and functionality of *luxS* demonstrated to date.	[77]
Photorhabdus luminescens	+	-	+	yes	nd	*lsr* locus is involved in specific interaction with nematode *H. bacteriophora*.	[56]
				yes	+/AI-2	*luxS* is positively involved in biofilm formation, oxidative stress resistence and early steps of insect invasion and negatively controls twitching mobility.	[78]
Rhodobacter capsulatus	-	-	+	nd	nd	*lsrB* is present, status of other genes unknown (sequencing not complete).	This work
Rhodobacter sphaeroides 2.4.1	-	-	+	nd	nd	Lsr-receptor is present but not LuxS.	This work
Salmonella enterica	+	-	+	yes	nd	*luxS* needed for fitness in chickens but not on cilantro leafs.	[79]
Salmonella typhimurium	+	-	+	yes	+/AI-2	Expression of Lsr ABC-transporter needed for AI-2 uptake.	[20]

				yes	+/AI-2	Lsr-mediated AI-2 transport and processing demonstrated.	[22]
Marinomonas sp. MED121	-	(+)	-	nd	nd	Unfinished sequence. Homolog of LuxPQ-receptor found.	This work
Neptuniibacter caesariensis	-	(+)	-	nd	nd	Potential hmolog of LuxPQ-receptor found.	This work
Sinorhizobium meliloti	-	-	+	nd	nd	Lsr receptor complex is present on the pSymB-plasmide.	This work
Shigella dysenteriae	+	-	+	nd	nd	No studies about QS-2 to date.	-
Shigella flexneri	+	-	+	yes	+/SN	Expression of *virB* depressed in *luxS* mutant and enhanced by AI-2 conditioned supernatants.	[80]
Vibrio anguillarum	+	+	-	yes	nd	The function of QS-2 (VanS, VanPQ) is redundant to QS-1 (VanMN)	[81]
Vibrio cholerae	+	+	-	yes	+/SN	Regulation of virulence together with CAI-1 quorum sensing system.	[82]
Vibrio harveyi	+	+	-	yes	+/SN	Regulation of luminescence.	[83]
Vibrio fischeri	+	+	-	yes	+/SN	Regulation of luminescence and host colonisation.	[84]
Vibrio parahaemolyticus	+	+	-	yes	+/SN	Regulation of T3SS activity.	[85]
Vibrio vulnificus	+	+	-	yes	+/SN	Coordination of the expression of virulence factors.	[38]
Yersinia pestis	+	-	+	nd	nd	QS not involved in biofilm formation.	[86]
				nd	nd	Only functionality of *luxS* and AI-2 production demonstrated. No loss of pathogenicity toward mice in *luxS* mutant	[87]
**b. Bacterial species without a *luxP*- or *lsrB*-gene homolog in their genome for which a metabolic role was hypothesized**							
Actinobacillus pleuropneumoniae	+	-	-	none	-/SN	*luxS* mutant shows attenuated virulence, that is not complemented by other bacteria within the same pool with a still intact gene: metabolic role postulated.	[88]
Borrelia burgdorferi	+	-	-	C	-/SN	AI-2 supernatants modulate protein expression.	[89]
				C	nd	It is speculated that RbsB may serve as the AI-2 receptor.	[90]
				C	+/DPD	Lacks complete AMC (missing MetE and MetH) and responds to DPD which regulates the expression of outer surface lipoprotein VlsE.	[91]
				none	nd	*luxS* mutant retains infectivity in mice and AI-2-like activity could not be detected in culture supernatants or concentrated cell lysates.	[92]
				none	nd	Major, if not sole, function for LuxS is metabolic: not required for tick colonization, transmission to mammalian host or disease induction.	[41]
Clostridium difficile	+	-	-	H	-/AI-2	RolA is a negative regulator of LuxS, but exogenous AI-2 has no effect on toxin production. Role of *luxS* can not be conclusively determined.	[93]
				C	+/SN	Upregulation of *tcd*-transcript levels by *luxS*-conditioned supernatants.	[94]
Erwinia amylovora	+	-	-	none	-/SN	Effects in *luxS*-mutation are to ascribe to disrupted a AMC, co-cultivation with wildtype does not relieve mutant phenotype.	[95]
				C	nd	Only *luxS* functionality shown. No assessment of phenotype or AI-2 response, yet presence of QS-2 claimed.	[96]
Helicobacter pylori	+	-	-	C	nd	Only AI-2 production shown, only genetic complementation	[97]

						performed.	
				C	nd	*luxS* is responsible for growth-phase dependent expression of *flaA*, genetic but not chemical complementation performed.	[98]
				none	nd	AI-2 is not involved in modulating virulence factors in *H. pylori* and 2D protein profile of *luxS* mutant is identical to the one of the wildtype.	[99]
				C	nd	*luxS* is important for motility and infectivity on gerbils, no chemical complementation.	[100]
				none	nd	AI-2 is by-product of AMC, phenotype result of metabolism not QS.	[101]
Lactobacillus rhamnosus	+	-	- [d]	none	-/DPD	Metabolic role (not QS) is important for growth and biofilm formation.	[102]
Listeria monocytogenes	+	-	-	none	-/SN	Enhanced biofilm formation in *luxS* mutant.	[103]
				none	-/AI-2	SRH, but not AI-2, has an effect on biofilm formation.	[104]
Neisseria meningiditis	+	-	-	C	nd	Bacteremic infection, no *luxS*- or AI-2 complementation	[105]
				none	-/AI-2	No AI-2 response	[106]
Proteus mirabilis	+	-	-	none	nd	No effect on motility, swarmer cell differentiation or virulence.	[107]
Serratia plymuthica	+	-	-	none	nd	No QS related function, just a metabolic role.	[108]
Staphylococcus aureus	+	-	-	none	-/SN	Impaired growth of *luxS* mutant under sulphur limitation, not relieved in coculture experiments: role is metabolic and is not related to quorum sensing.	[109]
Streptococcus mutans	+	-	-	C	+/AI-2	59 genes responsive to AI-2 identified by global transcriptome analysis	[50]
				C	+/SN	*luxS* positively affects biofilm formation, chemical complementation by different bacterial supernatants.	[49]
				C	nd	*luxS* affects on biofilm formation, no complementation.	[110]
				none	nd	No effect on biofilm formation.	[111]
Streptococcus pyogenes	+	-	-	none	-/SN	No auto-regulation of *luxS* by AI-2, no effect on virulence factors.	[112]
				C	nd	*luxS* regulates expression of virulence factors associated with epithelial cell internalization, no complementation performed.	[113]
				C	nd	Overexpression of virulence factors in *luxS* mutant, media dependent growth effect. No chemical complementation.	[114]
Vibrio angustum	+	-	-	none	nd	Bacterial supernatant does not induce luminescence in *V. harveyi* BB170.	[39]
**c. Bacterial species without a *luxP*- or *lsrB*-gene homolog in their genome for which QS-2 was claimed**							
Actinomyces naeslundii	+	-	-	C	+/DPD	Biofilm growth is dependent upon production of AI-2 by *S. oralis*, genetic complementation and chemical complementation using AI-2.	[115]
Bacillus subtilis	+	-	-	C	+/SN	*luxS* activity required for swarming and biofilm and aerial colonies	[116]

						formation, AI-2 containing supernatants negatively regulate *luxS*.	
Bifidobacterium adolescentis	+	-	-	nd	nd	Carries both LuxS and SahH pathway for recycling of SAH. No studies about QS-2 to date.	This work
Bifidobacterium longum	+	-	-	nd	nd	Carries both LuxS and SahH pathway for recycling of SAH. No studies about QS-2 to date.	[11]
Campylobacter jejuni	+	-	-	C	nd	*luxS* positively regulates motility, but no *luxS*- or AI-2 complementation.	[117]
				C	nd	*luxS* positively regulates *flaA*-expression and motility, but flagellar structure is unaffected, no complementation.	[118]
Clostridium perfringens	+	-	-	C	+/SN	Reduced production of toxins by *luxS* mutant. AMC genes not involved. Complemented by SN.	[119]
Enterococcus faecalis	+	-	-	nd	nd	Contains a *luxS* homolog, but it may be AI-3 that is involved in QS.	[120]
Pectobacterium carotovora	+	-	-	C	nd	Extracellular virulence factor expression is affected in *luxS* mutant. No genetic or chemical complementation.	[121]
				C	nd	LuxS-dependent signalling modulates the expression of pectinolytic enzymes. No genetic or chemical complementation.	[122]
Escherichia blattae	+	-	nd	nd	nd	Carries both LuxS and SahH pathway for recycling of SAH. No studies about QS-2 to date.	[43]
Photobacterium profundum	+	-	-	nd	nd	Belongs to Vibrionaceae and has a *luxS* gene, but does not have *luxPQ*.	[123]
Porphyromonas gingivalis	+	-	-	C	nd	Differential expression of hemin-acquisition genes, no complementation.	[124]
				C	nd	*luxS* mutant shows reduced protease and haemagglutinin activities. No chemical complementation.	[125]
				C	nd	*S. gordonii luxS* mutant is unable to form biofilm with *P. gingivalis*	[46]
				C	+/SN	Upregulation of stress genes in mutant, partial complementation with SN.	[126]
				C	+/SN	Regulation of genes coding for hemin and iron acquisition systems, complementation with AI-2 supernatants.	[127]
Pseudomonas aeruginosa	-	-	-	C	+/SN	A number of genes is induced by AI-2 supernatants, even if the strain lacks both *lsrB* and *luxS*.	[128]
Serratia marcescens	+	-[e]	-[e]	C	+/SN	Strain-dependent impact on virulence and production of antibiotics, chemical complementation with SN.	[129]
Shewanella spp.	+	-	-	C	nd	Identification of *luxS* gene and *V. harveyi*-mediated detection of AI-2 in various *Shewanella* species.	[130]
Staphyoycoccus epidermis	+	-	-	C	+/SN	Mutant shows enhanced biofilm formation *in vitro* and virulence, chemical complementation performed with supernatants.	[131]
Streptococcus anginosus	+	-	-[f]	C	+/DPD	Antibiotic susceptibility altered in *luxS* mutant and restored by AI-2.	[132]
				C	Interference	Biofilm formation in *S. anginosus*, *S. intermedius* and *S. mutans.,* competitively inhibited by a synthetic bromated furanone.	[133]

Streptococcus gordonii	+	-	- [f]	C	nd	*luxS* mutant unaffected in growth and ability to form biofilms on polystyrene surfaces, but unable to form a mixed species biofilm with a LuxS-null strain of the periodontal pathogen *Porphyromonas gingivalis*.	[46]
Streptococcus pneumoniae	+	-	-	C	nd	*luxS* mutant less fit and outcompeted by wildtype, no complementation.	[134]

[a] +: positive (>50% protein identity); (+): positive (<50% protein identity); -: negative (<30% protein identity); nd: not determined;

[b] nd: not determined; C: claimed; H: hypothesized; Q: questioned;

[c] +: successful complementation; -: unsuccessful complementation; nd: not determined; AI-2: complementation with pure autoinducer; DPD: complementation with precursor; SN: complementation with co-culture or conditioned supernatants of *luxS*-positive strain;

[d] Hypothesized on the basis of the genomes of 47 fully sequenced Lactobacillales (all *lsrB*-negative).

[e] Hypothesized based on *Serratia proteomaculans* sequence;

[f] Hypothesized on the basis of more than 10 known *Streptococcus* complete genomes belonging to the species *S. pneumoniae*, *S. mutans, S. pyogenes*, *S. agalactiae*, *S. suis*, *S. equis* and *S. zooepidemicus*.
