# Supplementary material for: Lack of genomic evidence of AI-2 receptors suggests a non-quorum sensing role for luxS in most bacteria
Source: BMC Microbiol. 2008 Sep 20;8:154. doi: 10.1186/1471-2180-8-154 (PMC2561040; doi:10.1186/1471-2180-8-154)
Supplement: Additional file 2 — Figure 1s. Phylogenetic relationships among sequenced Bacillus species on the basis of complete luxS sequences and the presence of the lsrB receptor in the respective genomes. [file 1471-2180-8-154-S2.pdf]

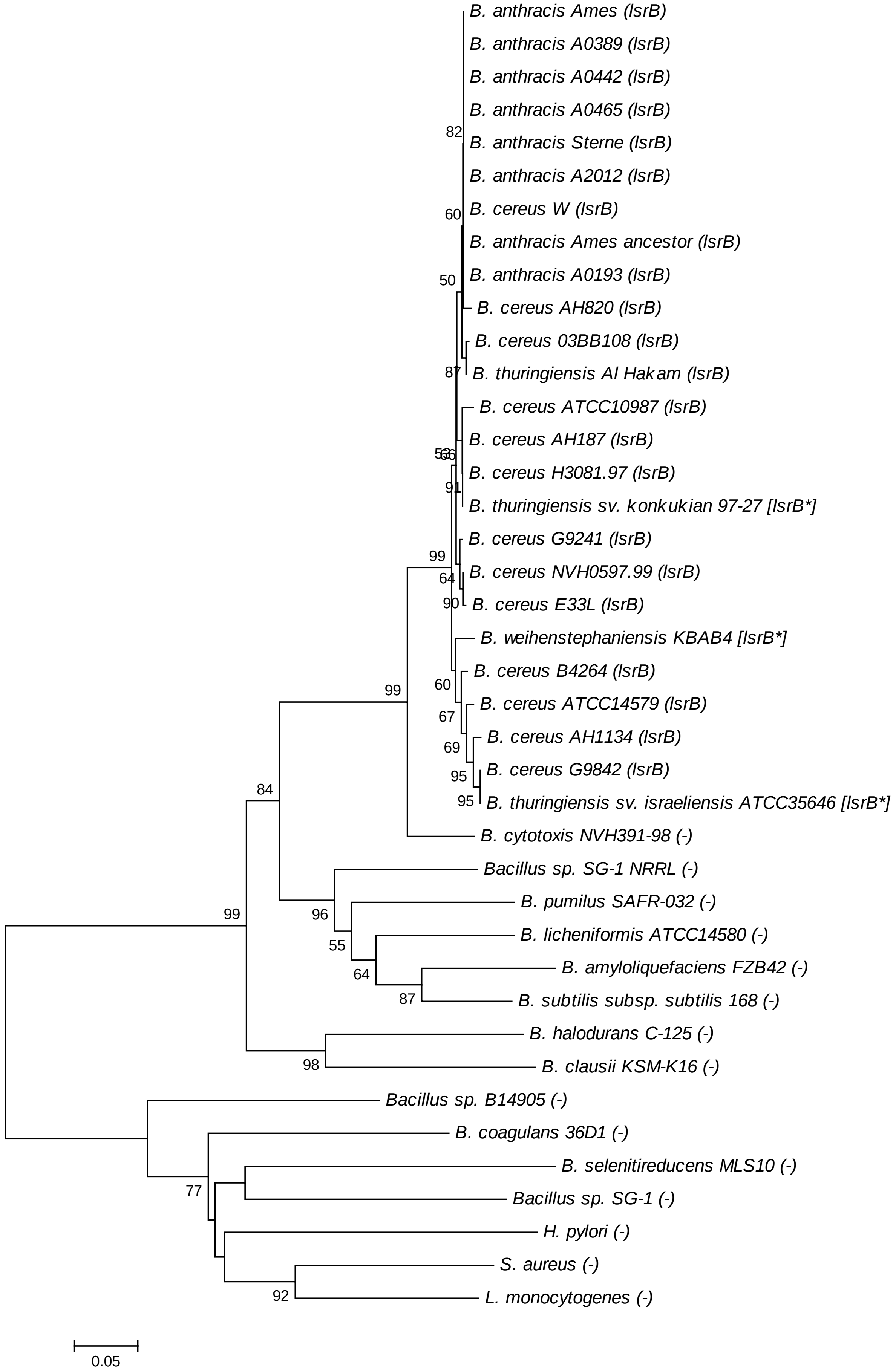
B. anthracis Ames (lsrB)
B. anthracis A0389 (lsrB)
B. anthracis A0442 (lsrB)
B. anthracis A0465 (lsrB)
B. anthracis Sterne (lsrB)
B. anthracis A2012 (lsrB)
B. cereus W (lsrB)
B. anthracis Ames ancestor (lsrB)
B. anthracis A0193 (lsrB)
B. cereus AH820 (lsrB)
B. cereus 03BB108 (lsrB)
B. thuringiensis Al Hakam (lsrB)
B. cereus ATCC10987 (lsrB)
B. cereus AH187 (lsrB)
B. cereus H3081.97 (lsrB)
B. thuringiensis sv. konkukian 97-27 [lsrB*]
B. cereus G9241 (lsrB)
B. cereus NVH0597.99 (lsrB)
B. cereus E33L (lsrB)
B. weihenstephaniensis KBAB4 [lsrB*]
B. cereus B4264 (lsrB)
B. cereus ATCC14579 (lsrB)
B. cereus AH1134 (lsrB)
B. cereus G9842 (lsrB)
B. thuringiensis sv. israeliensis ATCC35646 [lsrB*]
B. cytotoxis NVH391-98 (-)
Bacillus sp. SG-1 NRRL (-)
B. pumilus SAFR-032 (-)
B. licheniformis ATCC14580 (-)
B. amyloliquefaciens FZB42 (-)
B. subtilis subsp. subtilis 168 (-)
B. halodurans C-125 (-)
B. clausii KSM-K16 (-)
Bacillus sp. B14905 (-)
B. coagulans 36D1 (-)
B. selenitireducens MLS10 (-)
Bacillus sp. SG-1 (-)
H. pylori (-)
S. aureus (-)
L. monocytogenes (-)
0.05

Figure 1s. Phylogenetic relationships among sequenced Bacillus species on the basis of complete *luxS* sequences and presence of the *lsrB* receptor gene in the respective genomes. The distance tree was generated by the NJ method with the JC formula, without choosing any outgroup. Nodal supports were assessed by 1000 bootstrap replicates. Only bootstrap values greater than 50% are shown. The scale bar represents the number of substitutions per site. The presence of *lsrB* is indicated between parentheses. The asterisk shows the strains in which one or more genes encoding the Lsr-receptor complex present frameshift or rearrangement leading to truncated proteins. The *luxS* sequences of other Gram-positive bacteria *Helicobacter pylori* (NC_008086) , *Listeria monocytogenes* (NC_002973) and *Staphylococcus aureus* (NC_003923) were used as comparison. All genomes were retrieved at the NCBI database, accession numbers are: ***Bacillus amyloliquefaciens*** FZB42 (NC_009725); ***Bacillus anthracis*** str. Ames (NC_003997), Ames Ancestor (NC_007530), Sterne (NC_005945), A0193 (NZ_ABKF00000000), A0389 (NZ_ABLB00000000), A0442 (NZ_ABKG00000000), A2012 (NZ_AAAC00000000); ***Bacillus cereus*** str. 03BB108 (NZ_ABDM00000000), E33L (NC_006274), AH187 (NZ_AAUF00000000), AH820 (NZ_AAUE00000000), AH1134 (NZ_ABDA00000000), ATCC 10987 (NC_005707), ATCC 14579 (NC_004722), B4264 (NZ_ABDI00000000), G9241 (NZ_AAEK00000000), G9842 (NZ_ABDJ00000000), H3081.97 (NZ_ABDL00000000), NVH0597-99 (NZ_ABDK00000000), W (NZ_ABCZ00000000); ***Bacillus cereus* subsp. *cytotoxis*** NVH 391-98 (NC_009674); ***Bacillus clausii*** KSM-K16 (NC_006582); ***Bacillus coagulans*** 36D1 (NZ_AAWV00000000); ***Bacillus halodurans*** C-125 (NC_002570); ***Bacillus licheniformis*** ATCC 14580 (NC_006322); ***Bacillus pumilus*** SAFR-032 (NC_009848); ***Bacillus selenitireducens*** MLS10 (NZ_ABHZ00000000); ***Bacillus subtilis* subsp. *subtilis*** str. 168 (NC_000964); ***Bacillus thuringensis*** serovar *israelensis* ATCC 35646 (NZ_AAJM00000000), serovar *konkukian* str. 97-27 (NC_005957), Al Hakam (NC_008600); ***Bacillus weihenstephanensis*** KBAB4 (NC_010184); **Bacillus** sp. B14905 (NZ_AAXV00000000), NRRL B-14911 (NZ_AAOX00000000), SG-1 (NZ_ABCF00000000).
